# Evolution of a Core Gene Network for Skeletogenesis in Chordates

**DOI:** 10.1371/journal.pgen.1000025

**Published:** 2008-03-21

**Authors:** Jochen Hecht, Sigmar Stricker, Ulrike Wiecha, Asita Stiege, Georgia Panopoulou, Lars Podsiadlowski, Albert J. Poustka, Christoph Dieterich, Siegfried Ehrich, Julia Suvorova, Stefan Mundlos, Volkhard Seitz

**Affiliations:** 1 BCRT, Universitätsmedizin Charité, Berlin, Germany; 2 Max Planck Institute for Molecular Genetics, Berlin, Germany; 3 Department of Animal Systematics and Evolution, Free University, Berlin, Germany; 4 MPI for Developmental Biology Department 4 - Evolutionary Biology, Tübingen, Germany; 5 Bundesforschungsanstalt für Fischerei, Hamburg, Germany; 6 Institute for Medical Genetics, Charité, Universitätsmedizin Berlin, Berlin, Germany; University of Oxford, United Kingdom

## Abstract

The skeleton is one of the most important features for the reconstruction of vertebrate phylogeny but few data are available to understand its molecular origin. In mammals the *Runt* genes are central regulators of skeletogenesis. *Runx2* was shown to be essential for osteoblast differentiation, tooth development, and bone formation. Both *Runx2* and *Runx3* are essential for chondrocyte maturation. Furthermore, *Runx2* directly regulates *Indian hedgehog* expression, a master coordinator of skeletal development. To clarify the correlation of *Runt* gene evolution and the emergence of cartilage and bone in vertebrates, we cloned the *Runt* genes from hagfish as representative of jawless fish (*MgRunxA*, *MgRunxB*) and from dogfish as representative of jawed cartilaginous fish (*ScRunx1–3*). According to our phylogenetic reconstruction the stem species of chordates harboured a single *Runt* gene and thereafter *Runt* locus duplications occurred during early vertebrate evolution. All newly isolated *Runt* genes were expressed in cartilage according to quantitative PCR. In situ hybridisation confirmed high *MgRunxA* expression in hard cartilage of hagfish. In dogfish *ScRunx2* and *ScRunx3* were expressed in embryonal cartilage whereas all three *Runt* genes were detected in teeth and placoid scales. In cephalochordates (lancelets) *Runt*, *Hedgehog* and *SoxE* were strongly expressed in the gill bars and expression of *Runt* and *Hedgehog* was found in endo- as well as ectodermal cells. Furthermore we demonstrate that the lancelet Runt protein binds to *Runt* binding sites in the lancelet *Hedgehog* promoter and regulates its activity. Together, these results suggest that *Runt* and *Hedgehog* were part of a core gene network for cartilage formation, which was already active in the gill bars of the common ancestor of cephalochordates and vertebrates and diversified after *Runt* duplications had occurred during vertebrate evolution. The similarities in expression patterns of *Runt* genes support the view that teeth and placoid scales evolved from a homologous developmental module.

## Author Summary

Important molecular mechanisms underlying mammalian skeletogenesis have been described but knowledge about the evolutionary origin of these gene networks is limited. The *Runt* gene family (*Runx1–3*) is of extraordinary importance for skeletogenesis. *Runx2* deficient mice completely lack bone. *Runx2* and *Runx3* are essential for cartilage development and Runx2 regulates the key factor Indian hedgehog, which coordinates skeletogenesis. Here, we reconstructed Runt gene evolution in correlation to skeletal evolution. By analyzing lancelets, one of the closest living relatives of vertebrates, we revealed that the single *Runt* and *Hedgehog* family founder genes were co-expressed in primitive skeletal elements of the chordate stem species. Interestingly, at this stage the *Runt* and *Hedgehog* pathways were already directly linked to one another. Furthermore we isolated two *Runt* genes from a representative of jawless cartilaginous fish (hagfish) and three *Runt* genes from jawed cartilaginous fish (dogfish) which were all expressed in cartilage. The dogfish *Runt* genes were also found in teeth and placoid scales. This study suggests that *Runt* genes were involved in all ancient processes of chordate skeletogenesis. Furthermore the analysis supports the theory that most likely the gut was the tissue that originally secreted an acellular gill gut skeleton in the chordate ancestor.

## Introduction

The skeleton is a hallmark of vertebrates and has been widely used over the past decades for phylogenetic analyses [1]. However, little is known about its molecular evolution.

Descriptive data are available for the matrix proteins produced by the cells that constitute the skeleton in jawless vertebrates (epitomized by hagfish and lampreys, collectively termed agnathans). Beside species specific proteins [2] they possess cartilage with type II collagen (*Col2α1*), which is also the characteristic matrix protein for jawed vertebrates (gnathostomes) [3],[4]. Furthermore *Sox9*, which directly regulates *Col2α1* in mammals, was shown to be expressed in cartilage of the lamprey [3]. Interestingly *SoxE* (an invertebrate homolog to the mammalian *Sox8/9/10*) was found to be co-expressed with fibrillar collagen in the hemichordate *Saccoglossus bromophenolosu*s [5]. The expression was found in the pharyngeal endodermal cells, which are most likely responsible for the secretion of an acellular cartilage. Such an endodermal secretion was postulated to be primarily the ancestral mode of making pharyngeal cartilage in deuterostomes [5].

Up to now no *Runt* gene expression has been described in skeletal elements of lancelets, agnathans and jawed cartilaginous fish in spite of the fact that Runt transcription factors (*Runx1–3* synonyms: *Aml1–3/Cbfa1–3/Pebp2αa–c*) are central regulators of skeletal development in higher vertebrates [6],[7]. They are characterized by a highly conserved DNA binding Runt domain and the presence of two promoters [8]. Each *Runt* gene has two isoforms with different N-termini starting with a MASNS-like motif under the distal P1 promoter and a MRIPV sequence under the proximal Promoter P2. Furthermore the 3′ end has a conserved VWRPY-motif [8]. *Runx2* is indispensable for osteogenesis as mice bearing a homozygous mutation in *Runx2* completely lack bone [7], and *Runx2* is together with *Runx3* essential for cartilage differentiation [9],[10]. Futhermore Runx2 directly regulates the key signaling molecule *Indian hedehog* (*Ihh*), which coordinates cartilage differentiation, endochondral ossification and limb outgrowth [10]. From the three members belonging to the mammalian *Hedgehog* (*Hh*) family (*Ihh*, *Sonic hedgehog*, *Desert hedgehog*) also *Sonic hedgehog* (*Shh*) signaling is influenced by Runx2 during tooth morphogenesis [11]. *Runx2* haploinsufficiency causes the human bone disease cleidocranial dysplasia, further substantiating its importance for skeletal development [12]. Importantly, all three mammalian *Runt* genes are expressed in cartilage and have been shown to play a role in the formation and differentiation of skeletal elements [6],[10],[13]. Furthermore, all *Runt* genes in the mouse are involved in tooth formation [14].

In contrast to the extensively studied *Hox* genes, which are important for patterning, *Runt* genes are essential for features that represent evolutionary innovations of vertebrates such as bone [1]. Such innovations result from tinkering with existing processes, from the flexibility that arises from modifications to existing gene networks, and from selective advantage provided by gene duplications or modifications [15]. As simply as this theory explains an important evolutionary process, as difficult it is to functionally analyze how the genetic networks underlying innovations like the vertebrate skeleton evolved. Based on the central role of *Runt* genes for skeletogenesis in higher vertebrates we hypothesized that these genes played a role in the evolution of cartilage, bone and teeth and thus might be instrumental to understand skeletal evolution in chordates. We therefore analyzed number and expression of *Runt* genes in hagfish (*Myxine glutinosa*) as a representative of jawless vertebrates, in dogfish (*Scyliorhinus canicula*) as a representative of cartilaginous fish and lancelets (*Branchiostoma lanceolatum* and *B. floridae*) as representatives of celphalochordates to reconstruct if *Runt* genes were already expressed in the developing skeleton of the chordate, vertebrate and jawed vertebrate stem species. In addition, we tested if *Runt* and *Hh* are co-expressed in lancelets and if a functional interaction between the *Runt* and *Hh* pathways might have evolved before the cellular cartilage of vertebrates evolved.

In this study we show that the stem species of chordates harboured a single *Runt* gene, whereas three *Runt* genes were present before the emergence of gnathostomes. *Runt* genes are expressed in developing cartilage, teeth and placoid scales of cartilaginous fish and cartilage of jawless vertebrates. In adult lancelets the *Runt* gene is expressed together with *Hh*, in the endo- and ectoderm of the gill bars. Furthermore, we demonstrate that the lancelet Runt protein can directly bind to and activate the lancelet *Hh* promoter. This suggests that beside *SoxE* and fibrillar collagen two other key factors for vertebrate skeletogenesis (*Runt* and *Hh*) were part of an ancient gene network for skeletogenesis in the gill gut stabilizing the gill bars of the common ancestor of vertebrates and lancelets approximately 700 million years ago. Our finding that the gut is an ancient *Runt* expression domain of deuterostomes is in accordance with the hypothesis that endodermal secretion was the ancestral mode of making pharyngeal cartilage [5].

## Results

### Isolation of hagfish and dogfish *Runt* genes

We used a PCR-based approach using cDNA as well as genomic DNA to identify *Runt* genes in lower vertebrates. This led to the detection of two *Runt* genes in hagfish (*MgRunxA*, *MgRunxB*) and three *Runt* genes in dogfish (*ScRunx1–3*). All of these newly detected *Runt* genes had a 3′ end with the characteristic VWRPY-motif. The two different 5′ ends of the *Runt* genes amplified from embryonal dogfish cDNA were homologous to the 5′ mammalian promoter variant-1 (MASNS-like) and variant-2 (MRIPV-like) motifs, respectively. In the two hagfish *Runt* genes amplified from adult hagfish cDNA only a single 5′ gene end was detected. According to our Blast searches against the Ensembl pre-genome sequences of lamprey (*Pteromyzon marinus*) the two hagfish 5′ ends represent most likely the promoter variant 2. Because of the unavailability of hagfish embryos it could not be clarified if two Runt gene promoter 1 variants are expressed during early hagfish development.

Blast searches in whole genome databases (NCBI, JGI, Ensembl) revealed that there are most likely two Runt genes in the lamprey genome, and one *Runt* gene in cnidarians (*Nematostella vectensis*), nematodes (*Caenorhabditis elegans*), cephalochordates (*B. floridae*), and tunicates (*Ciona intestinalis*, *Oikopleura dioica*) [16],[17]. We detected two *Runt* genes in sea urchin (*Strongylocentrotus purpuratus*) [18],[19], which were located on the same genomic contig, two partial *Runt* genes in skate (*Raja eglanteria*) [20], three *Runt* genes in mammals [6],[7] and four in pufferfish (*Takifugu rubripes*) [21],[22] and also four in zebrafish (*Danio rerio*) including a duplicated *Runx2* gene [23]. In chicken (*Gallus gallus*) three *Runt* genes were found. An alignment of all newly detected *Runt* genes together with other deuterostome *Runt* genes is provided as supporting information (Figure S1) and the GeneBank accession numbers are given in the footnote.

### Conserved synteny of *Runt* and the *chloride intracellular channel* (*Clic*) genes in human, chicken and tunicate genomes

Comparable to the human *Runt* loci [24], the three orthologous chicken *Runt* genes are followed by a *Clic* gene on the complementary strand. The chicken *Runx1* on chromosome 1 is followed by a *Clic6* homologous gene, the chicken *Runx2* on chromosome 3 by a *Clic5* homologous gene and the chicken *Runx3* on chromosome 23 is followed by a *Clic4* homologous gene. In lancelet the *Runt* and *Clic* genes are located on different scaffolds (JGI assembly vers 1.0). However, in the genome of the tunicate *C. intestinalis* a *Clic* homologous gene was found in proximity to *Runt* on chr_12q (JGI, Assembly vers 2.0). This strongly suggests that the entire *Runt* locus was triplicated during the evolution of chordates.

### The last common ancestor of chordates harboured a single *Runt* gene

**Figure 1:**
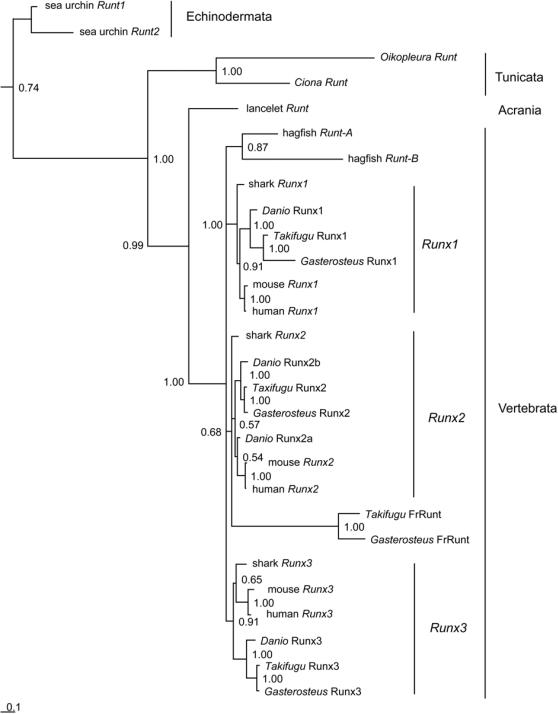
Phylogenetic tree (Bayesian inference) of chordate *Runt* genes. Numbers refer to branch support (Bayesian posterior probability) for the internal branches adjacent to the nodes. Sea urchin *Runt* genes were used to root the tree. Branch length reflects the number of substitutions per alignment site (compare scale bar).

**Figure 2:**
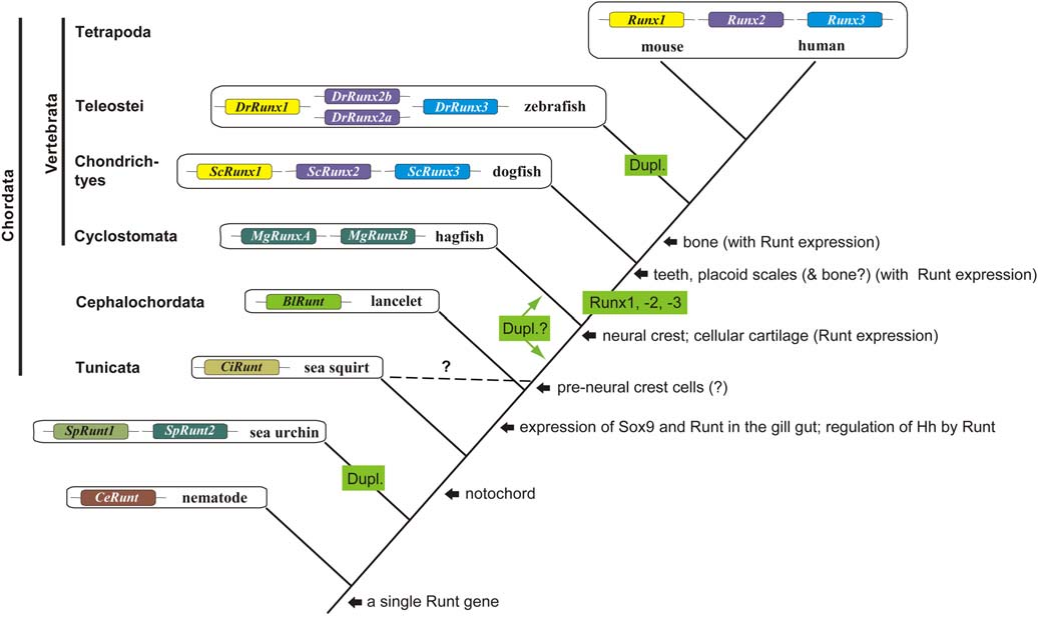
Overview of the *Runt* gene evolution *in* chordates. The stepwise evolution of cartilage and bone and the most likely time intervals of *Runt* gene duplications (Dup) are indicated. The position of tunicates is contentious [31] which is indicated by a dashed line. In this context it is of interest that pre-neural crest cells have been observed in tunicates [60].

Our phylogenetic analysis (Figure 1) suggests that the stem species of chordates harboured a single *Runt* gene, whereas the last common ancestor of jawed vertebrates harboured three *Runt* genes. In addition our results indicate that the dogfish Sc*Runx1–3* genes are orthologous to the *Amniota Runx1–3* genes. In contrast to this, the two hagfish *Runt* genes did not cluster with any of the three paralogous *Runt* genes from higher vertebrates. As outlined in Figure 2 several lineage specific Runt gene duplications have occurred: (a) in the sea urchin lineage, (b) in the stem species of bony fish and (c) probably also in hagfish. But there is a need for further data e.g. from whole genome comparison, to determine if the two hagfish *Runt* genes are a result of a *Runt* gene duplication in the stem species of vertebrates or evolved by a separate gene duplication event in the hagfish lineage.

### *Runt* gene expression in skeletal elements of hagfish and dogfish

**Figure 3:**
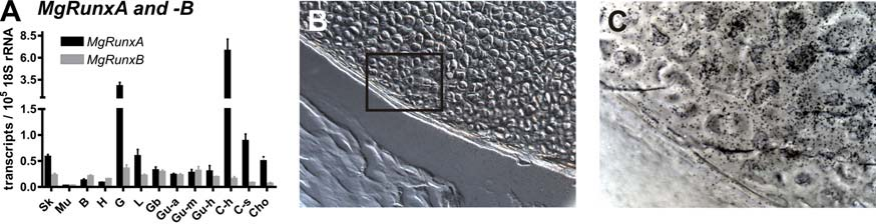
Analysis of hagfish *MgRunxA* and *–B* expression in different tissues of adult animals. Quantification of *MgRunxA* and *–B* expression by qRT-PCR (A). Whereas *MgRunxB* was only weakly expressed in all tissues analyzed, *MgRunxA* showed a strong expression in calcified cartilage gills and soft cartilage. Expression of *MgRunxA* was also detected by radioactive in situ hybridisation in hard cartilage tissue (B, C). Insert of (B) is shown at higher magnification in (C) displaying the silver grains of the autoradiography emulsion indicating *MgRunxA* expression. B: Brain, C-h: Hard cartilage, C-s: Soft cartilage, Cho: Chorda, G: Gills, Gb: Gall bladder, G-a: Anterior gut, G-m: Midgut, G-h: Hindgut, H: Heart, L: Liver, Mu: Muscle, Sk: Skin.

To determine a possible role for *Runt* genes in the skeleton we asked the question if *Runt* genes are expressed in skeletal elements of hagfish. Using quantitative Reverse Transcriptase PCR (qRT-PCR) from dissected tissues we found that the *MgRunxA* gene had its highest expression in hard cartilage, followed by the gill region and soft cartilage (Figure 3). Compared to the *MgRunxA* gene the *MgRunxB* gene was only weakly expressed with the strongest expression in the gill region. In situ hybridizations confirmed the high expression of *MgRunxA* in hard cartilage (Figure 3B and 3C).

**Figure 4:**
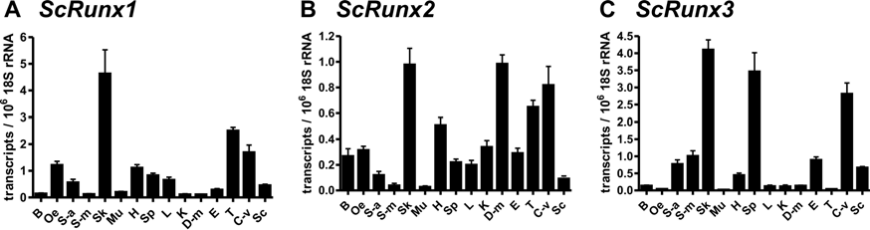
qRT-PCR results of *ScRunx1–3* expression. In dogfish the most prominent expression of all three *ScRunt* genes was in the skin. Also in visceral cartilage *ScRunx1–3* were strongly expressed. B: Brain, C-v: Visceral cartilage, D-m: Ductus mesonephric, E: Epididymis, H: Heart, K: Kidney, L: Liver, Mu: Muscle, Oe: Oesophagus, Sc: Spinal column, Sk: Skin, S-a: Anterior stomach, S-m: Middle part of stomach, Sp: Spleen, T: Testis.

**Figure 5:**
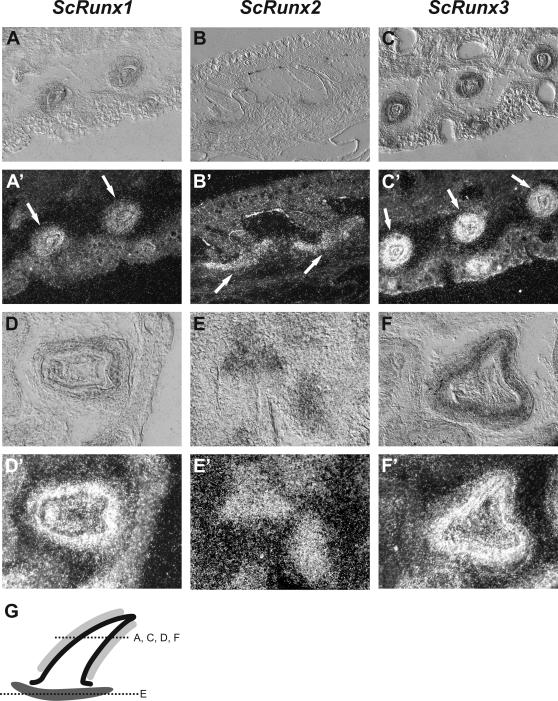
*ScRunx1–3* expression analysis by *in situ* hybridization in placoid scale (A–C) and tooth development (D–F). Bright field is given on top, dark field below. *ScRunx1* (A, D) and *–3* (C, F) are expressed in the basal epidermis cells of the stratum germinativum, which forms the enamel organ, whereas *ScRunx2* (B, E) is found at the site of the developing basal plate. These expression patterns were identical in teeth and placoid scales. (G) Scheme of *Runt* expression in placoid scales and teeth with overlapping expression of *ScRunx1* and –*3* in the stratum germinativum (light grey) and *ScRunx2* in the developing basal plate (dark grey). Dotted lines represent section planes of transverse sections in (A,C–F). Section in (B) is a longitudinal section.

**Figure 6:**
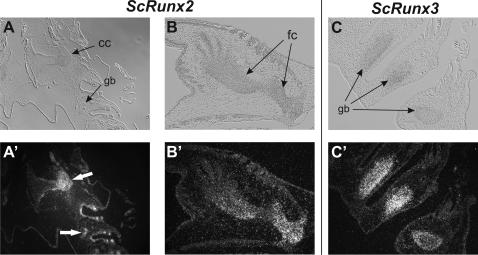
Expression of *ScRunx2* and *–3* in developing dogfish cartilage. Expression of *ScRunx2* was detected in developing cranial and gill bar cartilage (A) and in the proximal cartilage elements of the pectoral fin (B). Expression of *ScRunx3* was detected in developing visceral cartilage (C). Cc: cranial cartilage, gb: gill gut cartilage, fc: fin cartilage.

In adult dogfish the *Runt* genes show ubiquitous expression but it is noteworthy that all *Runt* genes had their third highest expression in the gill gut cartilage. For all three dogfish *Runt* genes the highest expression was found in the skin (Figure 4). We performed in situ hybridization to characterize the distribution of *Runt* expression in the skin (Figure 5A–5C). All three *Runt* genes were expressed in the placoid scales in the skin of dogfish embryos. *ScRunx1* and *ScRunx3* were expressed in the basal epidermis cells of the stratum germinativum, whereas *ScRunx2* was found at the site where later the basal plate will develop. Based on the similarities between scales and teeth we performed expression analysis of *Runt* genes in the developing teeth of dogfish embryos. In the developing teeth the same expression pattern of *ScRunx1–3* was found (Figure 5D–5F). *ScRunx1* and *ScRunx3* were expressed at a distal position and ScRunx2 was found at a basal position. Figure 5G shows a schematic of the different sites of *Runt* expression in teeth and placoid scales. In addition, *Runt* genes were also expressed in the developing skeleton. *Runx2* expression was detected in cranial cartilage and skeletal elements of the fin whereas *Runx2* and *Runx3* expression was found in gill gut cartilage (Figure 6).

### Expression of *Runt* during lancelet (*B. floridae*) development in the notochord, gut and neural tube

**Figure 7:**
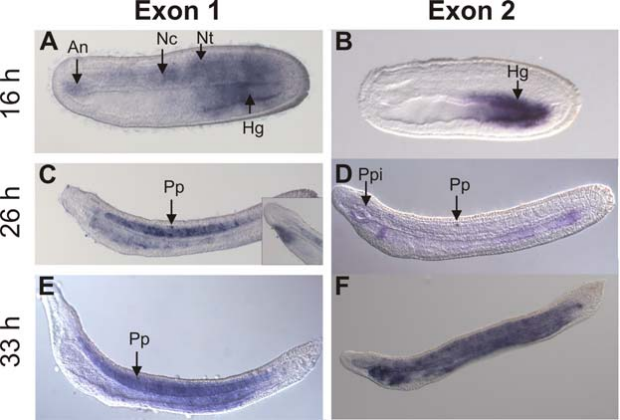
*Runt* gene expression in lancelet larvae (*B. floridae*). Anterior site is located to the left and the dorsal site towards the top. Whole mount in situ hybridization at stages of 16 h (A, B), 26 h (C, D) and 33 h (E, F). A), C) and E) *Runt* gene exon 1 variant. B), D) and F) *Runt* gene exon 2 variant. Note that the primary pigment spot, indicated by an arrow, lays in the nerve chord and does not represent a *Runt* expression domain. An: Anterior notochord, Nt: Neural tube, Nc: Notochord, Hg: Hindgut, Pp: Primary pigment spot, Ppi: Preoral pit.

To be able to reconstruct the *Runt* expression domains in the chordate stem species and to see if *Runt* was expressed in ancient skeletal elements such as the notochord, we analyzed *Runt* gene expression in lancelets, the putative sistergroup of vertebrates. Using whole mount in situ hybridization of early developmental stages (early and late gastrula) a diffuse *Runt* staining, indicating a maternal *Runt* expression, was detected, comparable to the description of maternal *Runx1*, *–2b*, and *–3* expression in zebrafish. [25]–[27]. Two different probes were used, corresponding to the *Runt* gene variant starting with exon 1 (transcribed from the distal promoter P1) and the *Runt* gene variant starting with exon 2 (transcribed from the proximal promoter P2). These two probes showed overlapping staining patterns (Figure 7).

The *Runt* gene variant P1 was expressed at the 8 somite stage (16 h) in the posterior part of the gut, the notochord and the developing neural tube (Figure 7A). At 26 h *Runt* expression can be predominantly seen in the middle part of the notochord, the midgut and foregut (Figure 7C). An inconsistent staining pattern was also detected at this stage in about 50% of the larvae immediately below the preoral pit (Figure 7C insert). At 33 h the larvae showed persistent expression of the *Runt* exon 1 variant in the notochord and neural tube, but also in the midgut region (Figure 7E).

The *Runt* gene variant P2 was exclusively expressed in the hindgut at 16 h (Figure 7B). At 26 h the expression domain extended throughout the entire gut and a signal was also found in a confined region of the foregut (Figure 7D). At 33 h *Runt* expression was found throughout the entire larvae with the most intense signals in the tailbud and in the anterior gut region. (Figure 7F).

### *Runt*, *SoxE* and *Hedgehog* expression in gill gut region of adult lancelet (*B. lanceolatum*)

**Figure 8:**
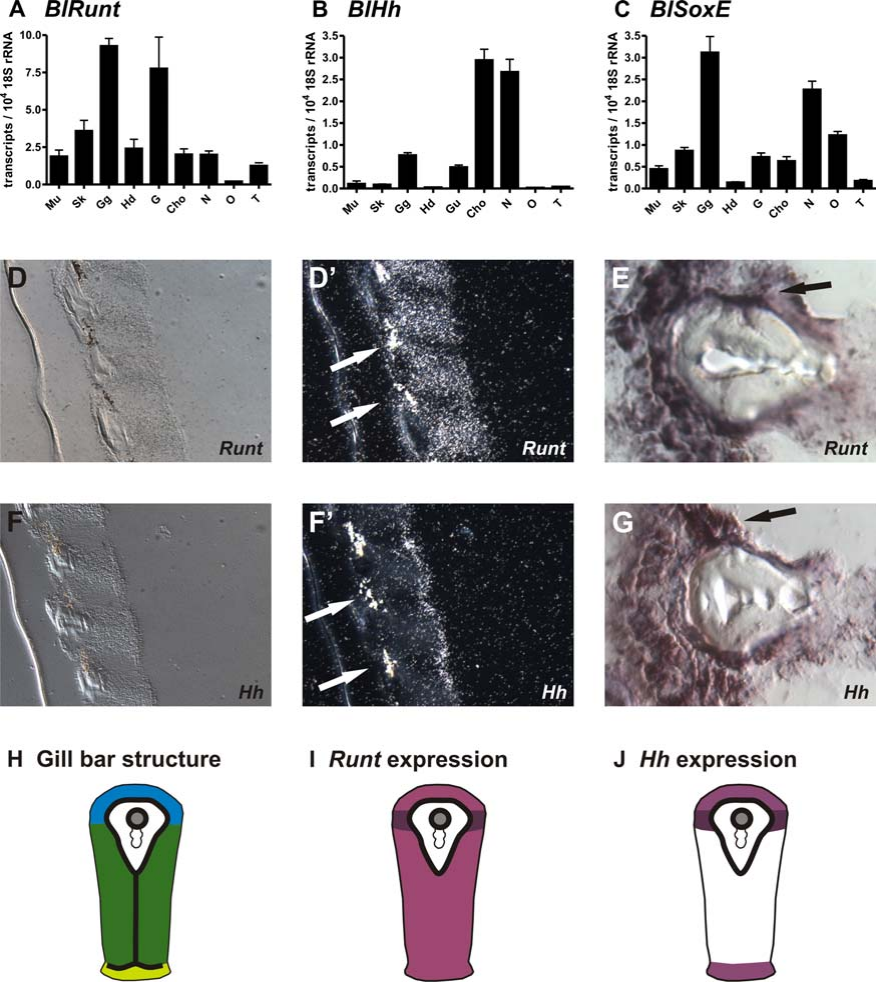
Analysis of *Runt*, *SoxE* and *Hh* gene expression in adult lancelet (*B. lanceolatum*). (A–C) Quantification of *Runt*, *SoxE* and *Hh* expression in different tissues. (A) The strongest *Runt* expression is seen in the gill gut region followed by the gut and skin. (B) *Hh* is most strongly expressed in the chorda and neural tube followed by the gill gut and gut. (C) *SoxE* has its strongest expression in the gill gut and neural tube. Mu: Muscle, Sk: Skin, Gg: Gill gut, Hd: Hepatic diverticulum, G: Gut, Cho: Chorda, N: Neural tube, O: Ovaries, T: Testis. (D–G) *in situ* hybridization for *BlRunt* and *BlHh* show high expression in the endoderm and ecotoderm of the gill bars. (D–E) *Runt* expression. (F–G) *Hh* expression. (D, F) Bright field images. (D′, F′) Dark field images of radioactive in situ hybridizations. (E, G) Non-radioactive in situ hybridizations. High expression of *BlRunt* and *BlHh* was found in a cell population between the endodermal epithelium with cilia and the ectodermal gland epithelium directly adjacent to both sites of the acellular matrix (arrows). (H–J) Schematic drawing of *Runt* and *Hh* expression sites in secondary gill bars as shown in (D–G). (H) The gill bar tissue consists of three different single layered epithelia attached to a basal membrane - atrial epithelium (blue), lateral epithelium (dark green) and pharyngeal epithelium (light green). The basal membrane is indicated by the bold black line. The skeletal rod of secondary gill bars contains a skeletal vessel (grey filled circle) that is formed by basal membranes, and does not contain endothelial cells. (I) *Runt* expression is found throughout the gill bar epithelia (light purple) with strongest expression adjacent to the skeletal rods (dark purple). (J) *Hh* is expressed at weaker levels in the atrial and pharyngeal epithelium (light purple) and at high levels in the cell population adjacent to the skeletal rods (dark purple).

Our analysis had shown that *Runt* genes are expressed in cartilaginous tissue of the hagfish as well as in the notochord of lancelets indicating a possible role in the development of the ancestral skeleton. We next asked the question, if *Runt* expression can be found in skeletal elements of adult lancelets. Based on the recent observation that adult lancelets express fibrillar collagen in their gill bars [5],[28] we hypothesized that the gill bars represent an ancestral form of cartilage regulated by similar pathways of chondrogenesis as in higher vertebrates. We showed previously that in adult lancelets only the *Runt* exon 2 variant is expressed [9]. As shown in Figure 8A, qRT-PCR demonstrated expression in almost all tissues, a finding that is in accordance with the broad staining pattern of the exon 2 *Runt* gene variant at 33 h PF (Figure 7F). However, the most intense signals in adult lancelets were found in the gill gut and the gut. Furthermore qRT-PCR showed that the lancelet *SoxE* gene had its highest expression and *Hh* its third highest expression in the gill gut region (Figure 8B and 8C). To determine where exactly *Runt* and *Hh* genes were expressed in the gill bars we performed in situ hybridization on tissue sections (Figure 8D–8G). We detected *Runt* and *Hh* gene expression in the endo- and ectodermal epithelial cells of primary and secondary gill bars (Figure 8D–8J) but not in the mesodermal coelomic cells of the primary gill bars (data not shown). Interestingly *Runt* and *Hh* were strongly coexpressed in a cell population between the endodermal epithelium with cilia and the ectodermal gland epithelium directly adjacent to both sites of the acellular matrix (arrows in Figure 8D–8G). The *Hh* signal was confirmed by immunohistochemistry (data not shown).

### Direct regulation of lancelet *Hedgehog* by Runt

**Figure 9:**
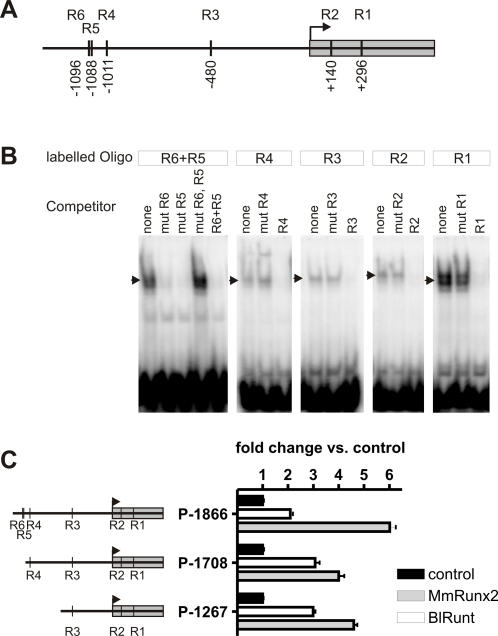
*Runt* dependent regulation of the *B. floridae Hh* promoter. (A) Scheme of the *BfHh* promoter with putative Runt binding sites. Number and position relative to the transcription start site is given. (B) Electrophoretic mobility shift assays using oligos containing R1–R6 Runt binding sites. BlRunt can bind to each of the putative binding sites. Strongest binding is observed for the oligo with the closely adjacent binding sites R5 and R6 and for the R1 oligo. Nuclear extracts without BlRunt do not show a mobility shift of the oligos (data not shown). (C) Runt dependent activation of the *BfHh* promoter in NIH3T3 cells. Overexpression of either BlRunt or MmRunx2 leads to activation of the indicated promoter constructs compared to constructs co-transfected with an empty expression vector.

As both *Runt* and *Hh* showed co-expression in the gill gut region, we analyzed whether a functional relationship between both genes, as it is known for the mouse [10], exists in lancelets. Analysis of the *B. floridae Hh* promoter revealed several putative Runt binding sites (Figure 9A). All of them were capable of binding to *B. lanceolatum* Runt as shown by electrophoretic mobility shift assays (Figure 9B). To provide further evidence for a Runt dependent regulation of *BfHh* we cloned different fragments of the *BfHh* promoter into the pGL3-basic luciferase reporter vector. Both, MmRunx2 and BlRunt were able to activate the different promoter constructs (Figure 9C).

## Discussion

### *Runt* gene evolution in chordates

In order to get insight into the molecular mechanisms underlying the evolution of the skeleton we analyzed the evolution of the *Runt* gene family in various representative species. *Runt* genes are important regulators of neurogenesis and hematopoiesis [29],[30] and they are essential for mammalian skeletogenesis [7],[10]. Our analysis revealed that the stem species of chordates harboured most likely only a single *Runt* gene and as outlined in Figure 2 independent *Runt* duplications occurred in the clades of sea urchin (*SpRunt1*, *SpRunt2*), and bony fish (duplication of *Runx2*). A chordate stem species with only a single *Runt* gene is the most parsimonious assumption since the genomes of cnidarians, nematodes, cephalochordates and tunicates harbour also only a single *Runt* gene. The presence of two *Runt* genes in sea urchin is most likely a result of a tandem duplication, as we found both genes on a single genomic contig and they cluster together in our phylogenetic analysis (Figure 1). It was recently postulated that tunicates and not cephalochordates are the sistergroup of vertebrates [31],[32]. Focusing on our aim to reconstruct the framework for *Runt* gene evolution, both alternative taxonomic positions of tunicates and lancelets would be consistent with our hypothesis that the stem species of chordates harboured only a single *Runt* gene.

In accordance with the evidence for at least one genome wide duplication, 350 to 650 million years ago [33],[34] we detected in dogfish (as a representative of the jawed cartilaginous fish) three *Runt* genes, orthologous to *Amniota Runx1*, *–2* and –*3* genes, whereas only two *Runt* genes (*MgRunxA* and *MgRunxB*) were identified in hagfish (as a representative of jawless vertebrates). The phylogenetic tree (Figure 1) identifies the *MgRunxA* and *MgRunxB* genes as being closely related to the *Runx1–3* genes. However, it is unknown if these evolved by a hagfish specific duplication or by a *Runt* gene duplication in the stem species of vertebrates. The phylogenetic analysis of the divergent *Runt* genes does not give satisfactory high support and a comparative analysis of the *Runt* gene loci will be needed to resolve this problem.

In the pufferfish (*T. rubripes*) genome, an enigmatic fourth *Runt* domain gene (*FrRunt*) was detected in addition to the orthologs of the *Runx1*, –*2* and –*3* genes, which appeared to represent either a pufferfish-specific fast evolving derivative of *Runx2* or a direct descendant of the ancestral chordate *Runt* gene [22]. According to our data it is unlikely that the *FrRunt* gene represents a direct descendent of the ancestral chordate *Runt* gene which evolved in parallel with the vertebrate *Runt* genes [22] since we did not detect a *FrRunt* orthologous gene in tunicates, lancelets, hagfish and dogfish. Instead our phylogenetic analysis (Figure 1) and a comparison of the genomic environment of the *FrRunt* locus with the genomes of other bony fish (supporting information Figure S2) suggests that the *FrRunt* gene represents a fast evolving *Runx2* orthologous gene. Such an accelerated evolution within duplicated genes is a common phenomenon [35].

Our findings that beside the human [24] also the chick and tunicate (*C. intestinalis*) *Runt* genes are followed by *Clic* genes together with the evidence that the *FrRunt* gene represents a fast evolving *Runx2* gene suggests that during chordate evolution the entire *Runt* locus was triplicated.

### *Runt* genes and the evolution of cartilage and bone in vertebrates

Cartilage has evolved multiple times in metazoa [1]. Here we focus on the vertebrate cellular cartilage expressing Col2*α*1 as the predominant matrix protein. Differentiation of this cartilage is regulated by a molecular network including Sox9, a transcription factor that directly regulates *Col2α1* expression [36]. Furthermore *Sox9* is a target of PTH related protein (PTHrP) that controls chondrocyte differentiation through a negative feedback loop with *Indian hedgehog* (*Ihh*). Runx2 in turn directly regulates *Ihh* [10]. Besides *Runx2* also *Runx1* and *Runx3* genes are expressed during murine and zebrafish cartilage formation. However *Runx2* and *Runx3* appear to be the most important *Runt* genes for skeletogenesis [6],[23].

In hagfish soft and hard cartilage can be distinguished [2] and a Col2α1-homologous protein is expressed only in soft cartilage [4]. It is unknown if a protein homologous to Col1α1 is expressed in hard cartilage as it is the case in mammalian bone. As shown in Figure 3 the hagfish *MgRunxB* gene is only weakly expressed in both types of cartilage. However, the *MgRunxA* gene had its strongest expression in hard cartilage and its third highest expression in soft cartilage (Figure 3A). We only analyzed tissues from adult hagfish of medium size (30–40 cm). The fact that hagfish grow up to a length of 80 cm suggests that the *Runt* gene expression in hagfish cartilage is also of importance for the growth of the skeleton.

The view that *Runt* genes have a conserved functional role in skeletogenesis is also supported by our finding of *Runt* gene expression in the developing cartilage of dogfish. We detected a strong expression in visceral cartilage for all three dogfish *Runt* genes by qRT-PCR (Figure 4). Furthermore we performed in situ hybridizations on dogfish embryos and found *ScRunx2* to be expressed in the cartilage of the fin and together with *ScRunx3* in the gill gut cartilage (Figure 6).

In lamprey (another representative of jawless vertebrates) the *Col2α1* gene is expressed in cartilage along with *Sox9* and *PTHrP*, indicating that they were already a part of the chondrogenic gene repertoire in early vertebrate evolution [3]. Our finding of dogfish and hagfish *Runt* expression in cartilage together with the well-known role of *Runt* genes in skeletogenesis, suggests that *Runt* genes can now be considered to be a part of the ancient molecular machinery for cartilage formation in the stem species of vertebrates.

### *ScRunx1–3* gene expression in teeth and placoid scales

Placoid scales are small conical structures in the skin of cartilaginous fish. We found that all three dogfish *Runt* genes are expressed in the developing placoid scales (Figure 5A–5C). Interestingly, the basal plate of scales and teeth is initiated by osteoblasts which continue to secrete bone matrix in a basal direction, while slightly later, the odontoblasts secrete dentine on the pulpar side on the basal plate [37]. Since Sc*Runx2* is expressed in the developing basal plate it is an interesting speculation that the expression of *Runx2* at this site might reflect the origin of bone as a dermal tissue in early vertebrate evolution. The dermoskeleton is the first to show mineralization in vertebrate phylogeny [38]. This mineralized dermoskeleton was composed of odontodes (dermal “teeth”) supported by extensively developed bone, imposing mineralization upon the collagenous layer of the dermis [38].

In placoid scales as well as in the developing teeth *ScRunx1* and *ScRunx3* were expressed in the stratum germinativum, whereas *ScRunx2* was found at the site where later the basal plate will develop (Figure 5A–5F). In mammals teeth develop as epithelial appendages in which sequential and reciprocal interaction between the ectoderm and underlying neural crest derived mesenchyme constitute a central developmental mechanism [1],[14]. The dental epithelial cells differentiate into ameloblasts and mesenchymal cells into odontoblasts, secreting the matrices enamel, and dentin respectively [1]. *Runx2* and *Runx3* expression is confined to mesenchymal tissues, whereas *Runx1* was found to be restricted to epithelia [14].

According to a classical view teeth evolved secondarily from skin denticles moving into the mouth (reviewed in [39]). However, this model was recently challenged by the proposal that sets of denticles on the pharyngeal (gill) arches and not external denticles were the precursors of the organized tooth families [39]. This alternative theory was based on the observations of homologous arrays of denticle whorls occurring within the pharyngeal region of jawless fish such as the thelodont *Loganellia* [40]. In this model the endoderm played an important role in the patterning process involved in the production of denticles on the postbranchial lamina [39]. It was assumed that the denticles on the postbranchial lamina have been formed in the presence of an inductive endoderm as one part of the internal visceral skeleton. This would be remarkably different to the development of external denticles, which are only under the influence of an inductive ectoderm [39].

Our *Runt* expression pattern supports the classical view that teeth and placoid scales have a common evolutionary origin, at least on the level of the molecular pathway underlying their development. In other words, the hypothesis that teeth and placoid scales evolved from a common developmental module, which might have been shifted and extended in its expression topology [41] is supported by the striking similarity of the *Runt* expression patterns in teeth and placoid scales.

### Conservation of molecular pathways in skeletogenesis

The gut appears to be an ancient expression domain of *Runt*. This expression in the chordate stem species can be reconstructed as *Runt* genes are expressed in the gut in representatives of the outgroup (sea urchins, nematode [16],[17] and the lancelet (this study, Figures 7 and 8). The *Runt* expression in the gill bars, structures that stabilize the gill gut, might be linked to the later role of *Runt* genes in the evolution of the pharyngeal skeleton. In zebrafish *Runx3* was shown to promote cartilage formation via the endodermal expression of *Runx3* in pharyngeal pouch cells [23].

However, in vertebrates most of the branchial arch cartilage, the cranial bone forming cells (osteoblasts), as well as the cells that deposit dentin (odontoblasts) are derived from the neural crest [42]. It was previously proposed that the neural crest acquired chondrogenic ability by recruiting proto-chondrogenic gene programs from the notochord, neural tube and gill gut [4], [5], [28], [43]–[45] Strikingly, we found high *Hh* expression together with high *Runt* expression in exactly these three sites indicating that the described interaction between the *Runt* and *Hh* pathways is of relevance for chordate cartilage evolution.

Whereas the homology of the gill gut in lancelets and vertebrates is well established [28] little is known about the molecular machinery necessary for development and maintenance of the skeletal-like structures of the pharyngeal gill slits in lancelets. The gill bars are stabilized by 15 nm thick filaments aligned parallel to the long axis of the rods, and are covered by a single layered epithelium, that can be morphologically distinguished into atrial, lateral and pharyngeal epithelium [46]. Gill bars gave a positive signal when stained with an antibody against type II collagen [28] indicating a cartilage-like structure, which appears to be acellular.

To get deeper insights into the molecular machinery underlying the early evolution of the skeleton we analyzed *Runt* and *SoxE* gene expression in adult lancelets. Our analyses revealed that both genes were highly expressed in the gill bar region (Figure 8). Furthermore our in situ hybridization results revealed that the lancelet *Runt* gene is expressed in atrial, lateral, and pharyngeal epithelium of ectodermal and endodermal origin (Figure 8D and 8E), but not in the mesodermal coelomic cells of the primary gill bars. It has recently been reported that the lancelet gills contains lymphocyte-like cells most likely located between the cells of the lateral and pharyngeal epithelia [47]. We cannot resolve these cells in our in situ hybridizations and thus cannot detect if *Runt* is expressed in these cells of the gill bars (Figure 8D and 8E). The finding of endodermal *Runt* expression supports the model in which endodermal secretion was the ancestral mode of making cartilage [5]. Since in deuterostomes the endoderm is a plesiomorphic *Runt* expression domain, *Runt* is likely to be present also in the endoderm of the gill gut in hemichordates.

Other crucial genes for mammalian skeletogenesis are *Ihh* and *Shh*. For *Ihh* a direct regulation by Runx2 has been shown and Runx2 influences *Shh* signaling in tooth development [10],[11]. Furthermore, *Runt* and *Ihh* genes are coexpressed during skeletogenesis in zebrafish [23],[25],[48]. We observed *Runt* expression in the midgut and foregut of lancelet larvae, similar to a recent study [49]. The exon 1 variant, however, showed additional expression in the notochord and neural tube (Figure 7A, 7C, and 7E). These expression domains were still detected in adult lancelets together with high *Hh* expression (Figure 8A and 8B). The observation that the single *Runt* and *Hh* genes of lancelets are co-expressed in the notochord, neural tube and in the adult lancelet gill gut (Figures 7 and 8 and reference [50]) prompted us to investigate if also the lancelet Runt protein might regulate lancelet *Hh* gene expression. In our *Hh* promoter studies the lancelet Runt protein bound directly to *Runt* binding sites in the lancelet *Hh* promoter and regulated the reporter gene driven by this promoter (Figure 9). The highest *Hh* expression together with *Runt* co-expression was found in the notochord, the neural tube, and the gill gut, all of which were previously proposed to be involved in the evolution of chordate cartilage [4],[5],[28],[44],[45]. It is thus likely, that the direct regulation of *Hh* by Runt was a relevant mechanism in chordate evolution. This suggests that the core gene network involved in vertebrate cartilage, bone and tooth formation was present prior to the divergence of cephalochordates and vertebrates and the duplication of the *Runt* and *Hh* genes.

Further research is needed to determine if a small cell group directly adjacent to both sites of the acellular matrix, with high *Runt* and *Hh* expression (arrows in Figure 8D–8G), is of special importance for cartilage formation in lancelets. Another interesting aspect will be to determine if a direct regulatory interaction between the *Runt* and *Hh* pathways is also present in hemichordates and whether a direct interaction between *Runt* and *Hh* pathways was maintained during vertebrate evolution in other important developmental processes, such as vertebrate hematopoiesis [29],[51].

## Materials and Methods

### Materials

Lancelets (*B. floridae*) were collected by shovel and sieve in water of 1 m in depth in Tampa Bay, Florida and in vitro fertilization, embryo culture and fixation were performed as previously described [52]. Adult *B. lanceolatum* were obtained from the Biologische Anstalt Helgoland. Hagfish (*M. glutinosa*) were collected by S.E. Material from adult dogfish (*S. canicula*) was obtained from the Biologische Anstalt Helgoland and dogfish embryos from the Aquazoo (Düsseldorf).

### Oligonucleotides

All primers and oligonucleotides employed in our study are given as supporting information. Primers for dogfish sequences can be found in Table S1. Primers for hagfish sequences are given in Table S2, and primers for amphioxus are listed in Table S3. Oligonucleotides employed for EMSAs are given in Table S4.

### Analysis of *Runt* gene sequence and number

Total RNA was isolated as described previously [17] from *B. floridae* (larvae), *B.lanceolatum* (adult), *M. glutinosa* (adult), *S. canicula* (embryos 4,5 cm, 6,5 cm, and 9,5 cm as well as adult animals). *Runt* genes were amplified by a strategy reported previously, using degenerated primers to amplify the conserved *Runt* domain followed by RACE PCRs to amplify the full length *Runt* genes [17]. The only exception was the amplification of the hagfish *MgRunxB* 5′ end which was obtained by inverse PCR with gene specific primers [53]

### Phylogenetic analysis

Alignments were obtained with ClustalW from 28 full length Runt amino acid sequences [54]. Ambiguously aligned proportions were omitted using Gblocks ver. 0.91b [55] with following parameters: minimum number of sequences for a conserved/flanking position (15/15), maximum number of contiguous nonconserved positions (8), minimum length of a block (5), allowed gap positions (all). The phylogenetic analysis was performed using MrBayes 3.1.5 [56], employing JTT+G+I as substitution model and running eight chains for 1.000.000 generations. Trees were sampled every 1000 generations and according to a saturation curve of likelihood values the first 500 trees were discarded as burn-in. Analysis was performed with *Runt* sequences from *O. dioica* (AAS21356.1), C. intestinalis (ci0100131551, ci010013155, ciad013o19, cinc013i02 and cies003n20), *B. lanceolatum* and *B. floridae* (AAN08567.1, AAN08565.1), *M. glutinosa* (DQ990008, DQ990009), *S. canicula* (DQ990010, DQ990012, DQ990014), *D. rerio* (NP_571678.1, AAS02047.1, AAQ88389.1, AAO85550.1). *T. rubripes* (BAF36011.1, BAF36001.1, AB280005.1, NP_001092121), *G. aculeatus* (Ensemble Gene Id: ENSGACP00000020145, ENSGACG00000012322, ENSGACG00000011721, ENSGACG00000007301), *M. musculus* (EDL03777.1, BAA03485.1, EDL29993.1) and *H. sapiens* (NP_001001890.1, EAX04278.1, NP_004341.1), while using the sea urchin *Runt* gene*s* from *S. purpuratus* (U41512.2, XM_776533.1) as an outgroup.

### In situ hybridizations

Whole mount in situ hybridizations with lancelet larvae were performed as previously described [43]. Radioactive in situ hybridizations on paraffin embedded tissue sections were performed as reported in [57] with the exception of using lower hybridization and washing temperatures of 50°C, and using 0,2× SSC instead of 2× SSC for washing of *B. lanceolatum* tissue sections. Non-radioactive in situ hybridization on cryo-sections of B. lanceolatum was carried out using the GenePaint System [58]. Probes for *MmIhh* and *ScRunx3* were used as hybridization controls for *B. lanceolatum*.

### Expression profiling of *Runt* genes in *M. glutinosa*, *S. canicula and B. lanceolatum* by qRT-PCR

QRT-PCR was performed on an ABIPrism 7900HT Cycler (Applied Biosystems, Forster City, USA) using SYBR Green PCR Master Mix (Applied Biosystems). TaqMan Reverse Transcription Reagents (Applied Biosystems) were used to synthesize the cDNA and primers were generated using the Primer Express software (Applied Biosystems). Quantification was performed using the standard curve method with dilutions of plasmids containing the sequence to be amplified in a known copy number as a standard. For the analysis of *SoxE* expression by qRT-PCR first a *SoxE* cDNA fragment was amplified by employing primers which were designed according to a *SoxE* sequence of *B. floridae*. Expression of target genes was normalized using 18S rRNA as reference.

### Immunohistology

For immunohistochemistry on paraffin sections citrate antigen retrieval was performed. Anti-human Ihh antibody (Santa Cruz) was applied 1∶50 over night. Secondary antibody (biotinylated anti-goat, Sigma-Aldrich) was applied 1∶500 for one hour. Subsequent staining was performed with the Vectastain ABC kit from Vector laboratories according to the manufacturers′ instructions.

### EMSA

Electrophoretic mobility shift assays for putative binding sites were performed as described in [59] with nuclear extracts from chicken DF-1 cells infected with a RCAS-virus expressing the *Runt* cDNA from *B. lanceolatum*. Specific binding was confirmed with a labeled oligo containing the putative binding site and using either wild type oligos or oligos with mutated binding sites as competitors.

### Luciferase reporter assays

PCR amplified fragments of the *B. floridae Hh* promoter (AC150424) were cloned into the pGL3-basic reporter vector. NIH3T3 cells were transfected in 24-well plates with the reporter constructs (250 ng per well) together with an expression vector containing either the cDNA for *BlRunt* or *MmRunx2* or an empty vector as control (100 ng per well). 5 ng per well of pRL-CMV were co-transfected for normalization. Cells were lysed with 100 µl passive lysis buffer (Dual Luciferase Assay Kit; Promega, Madison, USA). 5 µl of the lysate were measured using the Dual-Glo Luciferase Assay Kit (Promega) with 25 µl of the assay reagents each. Measurements were performed on a 1450 MicroBeta Scintillation and Luminescence Counter (Perkin Elmer, Waltham, USA). The result of a representative experiment is shown which was confirmed five times independently.

### Data deposition

The sequences reported in this paper have been deposited in the GenBank databases. Dogfish: MASNS-like-promoter variant 1, *ScRunx1* Acc-Nr DQ990011, *ScRunx2* DQ990013, *ScRunx3* DQ990015 and MRIPV-like-motifs promoter variant 2, *ScRunx1* DQ990010, *ScRunx2* DQ990012, *ScRunx3* DQ990014. Hagfish: *MgRunxA* DQ990008, *MgRunxB* DQ990009. Lancelet: *SoxE* EF051347.

## Supporting Information

Figure S1 Alignment used for Phylogenetic Analysis. Alignment (ClustalW, BioEdit: http://www.mbio.ncsu.edu/BioEdit/bioedit.html) of newly detected *Runt* genes in hagfish (*MgRunxA* and *B*, DQ990008, DQ990009) and dogfish (*ScRunx1*-3, DQ990010, DQ990012, DQ990014) with other deuterostome *Runt* genes. The conserved sequence blocks used for the phylogenetic analysis are underlined with #. Parameters used with Gblocks 0.91b were: Minimum number of sequences for a conserved / flanking position: 15/15; Maximum number of contiguous nonconserved positions: 8; minimum length of a block: 5; allowed gap positions: all. 338 (52%) of the original 645 alignment positions were used in the phylogenetic analysis.
Abbreviations: B.l.: *Branchiostoma lanceolatum*; C.i.: *Ciona intestinalis*; D.r.: *Danio rerio*; G.a.: *Gasterosteus aculeatus*; H.s.: *Homo sapiens*; M.m.: *Mus musculus*; M.g.: *Myxine glutinosa*; O.d.: *Oikopleura dioica*; S.p.: *Strongylocentrotus purpuratus*; S.c.: *Scyliorhinus canicula*; T.r.: *Takifugu rubripes*.
(0.09 MB DOC)

Figure S2 Synteny Analysis.
A search for cross-species conserved gene orders was performed as previously described [1]. We compared a larger contig of the *FrRunt* locus (Ensemble: Scaffold 39) than previously analyzed (Ensemble: Scaffold 835[2]) to the zebrafish genome and detected a synteny region between the 3′ genomic region of the *FrRunt* gene and chromosome 1 of zebrafish comprising *Fstl1* and *Gja5* (A). Furthermore we detected in the stickleback (G. aculeatus) genome a *FrRunt* orthologous gene with a genomic environment almost identical to the *FrRunt* gene locus (B). The gene orthologous to Clic 5 located 3′ of *Runx2a* in the zebrafish genome was found by Blast searches on group 1 in the stickleback genome. Together these results suggest that a translocation between a region of the 3′ end of the *FrRunt* locus and chromosome 1 had occurred in the common stem species of pufferfish and stickleback.
(0.05 MB DOC)

Table S1 Dogfish Primers.
Primers employed to amplify and analyze the expression of *Runt* genes in dogfish. PA: Primary amplification, RA: Reamplification.
(0.08 MB DOC)

Table S2 Hagfish Primers.
Primers employed to detect *Runt* genes and analyze *Runt* gene expression in hagfish. PA: Primary amplification, RA: Reamplification.
(0.07 MB DOC)

Table S3 Amphioxus Primers.
Primers employed to analyze *Sox9*, *Hedgehog* and *Runt* genes in lancelets.
(0.08 MB DOC)

Table S4 EMSA Oligos.
Oligos employed for the electrophoretic mobility shift assays.
(0.07 MB DOC)
