# Supplementary material for: Evolution of a Core Gene Network for Skeletogenesis in Chordates
Source: PLoS Genet. 2008 Mar 21;4(3):e1000025. doi: 10.1371/journal.pgen.1000025 (PMC2265531; doi:10.1371/journal.pgen.1000025)
Supplement: Figure S1 — Alignment used for Phylogenetic Analysis. Alignment (ClustalW, BioEdit: http://www.mbio.ncsu.edu/BioEdit/bioedit.html) of newly detected Runt genes in hagfish (MgRunxA and B, DQ990008, DQ990009) and dogfish (ScRunx1-3, DQ990010, DQ990012, DQ990014) with other deuterostome Runt genes. The conserved sequence blocks used for the phylogenetic analysis are underlined with #. Parameters used with Gblocks 0.91b were: Minimum number of sequences for a conserved / flanking position: 15/15; Maximum number of contiguous nonconserved positions: 8; minimum length of a block: 5; allowed gap positions: all. 338 (52%) of the original 645 alignment positions were used in the phylogenetic analysis. Abbreviations: B.l.: Branchiostoma lanceolatum; C.i.: Ciona intestinalis; D.r.: Danio rerio; G.a.: Gasterosteus aculeatus; H.s.: Homo sapiens; M.m.: Mus musculus; M.g.: Myxine glutinosa; O.d.: Oikopleura dioica; S.p.: Strongylocentrotus purpuratus; S.c.: Scyliorhinus canicula; T.r.: Takifugu rubripes. (0.09 MB DOC) [file pgen.1000025.s001.doc]

10 20 30 40 50 60

=========+=========+=========+=========+=========+=========+

S.p.1 MHITDVNVDHLLSSTAPLANHPSKDPVRRNNLHNSYKMAEGGQRNKASSV----------

S.p.2 -------------------------------------MTEVGQR-KGSSP----------

O.d. ---MLLNDSGLNFPQMDLSTSLLG----------------GNYYGSPFMYDNHNA-----

C.i. ---------------MSYTGSVSG----------------SQHTTS--------------

B.l. ----------MLEVYRWQAFDLDP--------------FRNSRRFSPFADPG--------

M.g.-RunxA --MHLPADTGHTRRFTPPSNTLTPN---------KLSDPHHHHHHHHHLHPHHHHHH---

M.g.-RunxB --------------------MWIS-------------DTPGRHHHSTQGGSVTTP-----

S.c.-Runx1 -----MASNSIFESFPSYQQCFMR-------------DTNTSRRFTPPSTTLSTG-----

S.c.-Runx2 -----MASNSIFSSVTPCQQNFFW-------------DPTTSRRFTPPSTTLPA------

S.c.-Runx3 -----MASNSIFDSFTTYPQSFLR-------------DPSTSRRFTPPSTTLTPSG----

D.r.-Runx1 ---------------MVFLWDAKY-------------EPAPGRRFTPPSTTLSSG-----

D.r.-Runx2b -----MASNSLFSSVTPCQQNFFW-------------DPSAGRRFSP------P------

D.r.-Runx2a -----MASNSLFSSVTPCQQNFFW-------------DPSTSRRFSPPSSSLQP------

D.r.-Runx3 -----MASNSIFDTFSSYSPSLLR-------------DPSTSRRFTPPSASFPCA-----

T.r.-Runx1 -----MASNSIFETVLSCHSALRK-------------DPAPGRRYTPPSTTLGSGG----

T.r.-Runx2 -----MASNSLFSTVTPCQQNFFW-------------DPSATRRFSPPSSSLQP------

T.r.-Runx3 -----MASNSIFDSLSSYSNSLLR-------------EPPTTRRFTPPSTSFPCS-----

T.r.-FrRunt -----MASNSLFSSTSP---LICW-------------DPVVKHRPMPSPGHHRME-----

G.a.-FrRunt -----MASNSLFSSTSP---MLYW-------------DPVVKHQPTPLPTNRHPD-----

G.a.-Runx1 -------------------RSLT--------------DPAPGRRYTPPSTTLASGG----

G.a.-Runx2 -----MASNSLFSTVTPCQQNFFW-------------DPSASRRFSPPSNSLQP------

G.a.-Runx3 -----MASNSIFDSFSNYSSSLLRG------------EPPTTRRFTPPSTSFPC------

M.m.-Runx2 -----MASNSLFSAVTPCQQSFFW-------------DPSTSRRFSPPSSSLQPGKMSDV

M.m.-Runx3 -----MASNSIFDSFPNYTPTFIR-------------DPSTSRRFTPPSTAFPCGGG---

M.m.-Runx1 -----MASDSIFESFPSYPQCFMR-------------DASTSRRFTPPSTALSPG-----

H.s.-Runx1 -----MASDSIFESFPSYPQCFMRECILGMNPSRDVHDASTSRRFTPPSTALSPG-----

H.S.-Runx2 -----MASNSLFSTVTPCQQNFFW-------------DPSTSRRFSPPSSSLQPGKMSDV

H.S.-Runx3 -----MASNSIFDSFPTYSPTFIR-------------DPSTSRRFTPPSPAFPCGGG---

######### ############

70 80 90 100 110 120

=========+=========+=========+=========+=========+=========+

S.p.1 -----------------------------------------------------------F

S.p.2 -----------------------------------------------------------C

O.d. ------------------------------------------------------------

C.i. ------------------------------------------------------------

B.l. ------KMGDPHRKVHPHFKG---------------------------------------

M.g.-RunxA ----HHQQQHQQQQASGAQQGE--------------------------------GALGKL

M.g.-RunxB --------GPGGGKAGEEASKS---------------------------------RGRRL

S.c.-Runx1 ------KMSDSLPLA--SPE---------------------------------SALS-KL

S.c.-Runx2 ----SGKMSDVSGMIPHQEPGPGVG---------------------TAGAAAALGRSLIR

S.c.-Runx3 ------KMSEP-VSLSQPTPAG---------------------------------LS-RI

D.r.-Runx1 ------KMSEGLPLGAQESTG--------------------------------AALVGKL

D.r.-Runx2b ---VSVKMNDVNSNAGPQQQQD-----------------------------GAVVPRLR-

D.r.-Runx2a ---VAGKMSDAQPEPN------------------------------------AVVPRLR-

D.r.-Runx3 ------KVTESQ-SMSAPGP-----------------------------------LRSGR

T.r.-Runx1 ------KMAEALPLGAQEAGGG-------------------------------GALMGKL

T.r.-Runx2 ---VPGKMNDVSSPTG--QPDA-----------------------------AAAVPRLR-

T.r.-Runx3 ------KIGDSNGAMATPGP-----------------------------------LRSRP

T.r.-FrRunt ------------------------------------------------------------

G.a.-FrRunt ------------------------------------------------------------

G.a.-Runx1 ------KMAEALPLGTQEAGG--------------------------------ASLVGKL

G.a.-Runx2 ---VSGKMNDVSSPTG--QPD------------------------------AAAVPRLR-

G.a.-Runx3 ------KLGDGNGAMATPGP-----------------------------------LRSRS

M.m.-Runx2 SPVVAAQQQQQQQQQQQQQQQQQQQQQQQQQQQQQEAAAAAAAAAAAAAAAAAAVPRLRP

M.m.-Runx3 ---GGGKMGENSGALSAQATAG---------------------------------PGGRT

M.m.-Runx1 ------KMSEALPLG--APDGG-------------------------------AALASKL

H.s.-Runx1 ------KMSEALPLG--APDAG-------------------------------AALAGKL

H.S.-Runx2 SPVVAAQQQQQQQQQQQQQQQQQQQQQQQE-------AAAAAAAAAAAAAAAAAVPRLRP

H.S.-Runx3 ---GG-KMGENSGALSAQAAVG---------------------------------PGGRA

130 140 150 160 170 180

=========+=========+=========+=========+=========+=========+

S.p.1 KGGERSIVDALSEYPG--ELVKTESPNFACSVLPNHWRCNKSLPVAFKVVSLG--ETKDG

S.p.2 KGGERSLVDALSDYPG--ELVKTESPNFICSLLPPHWRSNKSLPVAFKVVSLG--ETKDG

O.d. HLLRHGCIPGMEQHHGHTEMTKTDSPNFTCTQLPVHWRKNKSLPAPFKIVAMDPITVPDG

C.i. ---GRHVVDILSDHQG--ELIKTDSPNFLCTPLPQHWRVNKSLQTPFKVVALS--DIPDG

B.l. ---DRGLVDALADHPG--ELVRTDSPNFVCSVLPSHWRCNKTLPVPFKVVALG--DIPDG

M.g.-RunxA RPGDRPMVEVLADHPG--ELVRTDSPNFLCSVLPSHWRCNKTLPVAFRVVALG--DVADG

M.g.-RunxB GSVECVPIDILAEHAA--ELVRTDSPGILCSVLPSHWRCNKTLPVPFKVLCLV--DVPDG

S.c.-Runx1 RATDRSMVDVLADHPG--ELVRTDSPNFLCSVLPTHWRCNKTLPIAFKVVALG--DVPDG

S.c.-Runx2 PHENRTMVDIIADHPA--ELVRTDSPNFLCSVLPSHWRCNKTLPVAFKVVALG--DVPDG

S.c.-Runx3 RPETRTVVDVLQDHAG--ELVRTDSPNFLCSVLPSHWRCNKTLPVAFKVVALG--DVPDG

D.r.-Runx1 RMADRSMVEVLSDHPG--ELVRTDSPNFLCSVLPTHWRCNKTLPIAFKVVALG--DIPDG

D.r.-Runx2b AQENRSMAEIIADHPA--ELVRTDSPNFLCSVLPSHWRCNKTLPVAFKVVALG--EVPDG

D.r.-Runx2a PHDNRTMVEIIADHPA--ELVRTDSPNFLCSVLPSHWRCNKTLPVAFKVVALG--DVPDG

D.r.-Runx3 PVESRSVVDVLADHAG--ELVRTDSPNFLCSVLPSHWRCNKTLPVAFKVVALG--DVPDG

T.r.-Runx1 RMADRSMMEVISDHPG--ELVKTDSPNFLCSVLPTHWRCNKTLPIAFKVVALG--DIPDG

T.r.-Runx2 PHENRSMAEIIADHPA--ELVRTDSPNFLCSVLPSHWRCNKTLPVAFKVVALG--DIPDG

T.r.-Runx3 --DTRNMVDVLADHAG--ELVRTDSPNFLCSVLPSHWRCNKTLPVAFKVVALG--DVPDG

T.r.-FrRunt NQDVGTIQQPRMGIPR--GLVQTDSPNFLCTSLPQHWRCNKTLPRPFTVFALGN-DVPDG

G.a.-FrRunt DHDAKSRLQRSTAPPR--GLMQTDSPNFLCSSLPQHWRCNKTLPRAFTVVAVGN-DVPDG

G.a.-Runx1 RMADRGMVEVISDHPS--ELMKTDSPNFLCSVLPTHWRCNKTLPIAFKVVALG--DTPDG

G.a.-Runx2 PHENRSMAEIIADHPA--ELVRTDSPNFLCSVLPSHWRCNKTLPVAFKVVALG--DISDG

G.a.-Runx3 --DTRNVVDVLADHAG--ELVRTDSPNFLCSVLPSHWRCNKTLPVAFKVVALG--DVPDG

M.m.-Runx2 PHDNRTMVEIIADHPA--ELVRTDSPNFLCSVLPSHWRCNKTLPVAFKVVALG--EVPDG

M.m.-Runx3 RPEVRSMVDVLADHAG--ELVRTDSPNFLCSVLPSHWRCNKTLPVAFKVVALG--DVPDG

M.m.-Runx1 RSGDRSMVEVLADHPG--ELVRTDSPNFLCSVLPTHWRCNKTLPIAFKVVALG--DVPDG

H.s.-Runx1 RSGDRSMVEVLADHPG--ELVRTDSPNFLCSVLPTHWRCNKTLPIAFKVVALG--DVPDG

H.S.-Runx2 PHDNRTMVEIIADHPA--ELVRTDSPNFLCSVLPSHWRCNKTLPVAFKVVALG--EVPDG

H.S.-Runx3 RPEVRSMVDVLADHAG--ELVRTDSPNFLCSVLPSHWRCNKTLPVAFKVVALG--DVPDG

########################################################

190 200 210 220 230 240

=========+=========+=========+=========+=========+=========+

S.p.1 TMVTIAAGNDENYCAELKNNTAVMKNQVARFNDLRFVGRSGRGKSFTLSIFIYTNPPQIA

S.p.2 TVVTIGAGNDENCCAELKNNIAVMKNQVARFNDLRFVGKSGRGKSFTLSIFVYTNPPQIA

O.d. TQVTIYAGNDEEHSAELRNNTTTFKNNVARFNDLRFIGRSGRGKTFNLTLMVATNPPQIA

C.i. TTVTVMAGNDENYSAELRNASATMKGCVARFNDLRFLGRSGRGKSFNLTITIFSSPPQVA

B.l. TLVTVMAGNDENYSAELRNNQAVMKNQVARFNDLRFVGRSGRGKSFTLTITVFTSPPQVA

M.g.-RunxA TMVTVMAGNDENYSAELRNASAVIKNQVARFNDLRFVGRSGRGKSFTLTITVFSNPPQVA

M.g.-RunxB TEVGVLAGNDENCTAELRNTRAVTKERVARFNDLRFVGRSGRGKSLTLTITVFTRPPQVA

S.c.-Runx1 TLVTVLAGNDENYSAELRNATAVMKNQVARFNDLRFVGRSGRGKSFTLTITVFTNPPQVA

S.c.-Runx2 TVVTVMAGNDENYSAELRNASAVMKNQVARFNDLRFVGRSGRGKSFTLTITVFTNPPQVA

S.c.-Runx3 TLVTVMAGNDENYSAELRNASAVMKNTVARFNDLRFVGRSGRGKSFTLTITVFTSPPQVA

D.r.-Runx1 TLVTVMAGNDENYSAELRNATAAIKNQVARFNDLRFVGRSGRGKSFTLTITVFTNPPQVA

D.r.-Runx2b TVVTVMAGNDENYSAELRNASGVMKNQVARFNDLRFVGRSGRGKSFTLTITVFTNPPQVA

D.r.-Runx2a TVVTVMAGNDENYSAELRNASAVMKNQVARFNDLRFVGRSGRGKSFTLTITVFTNPPQVA

D.r.-Runx3 TLVTVMAGNDENYSAELRNASAVMKNQVARFNDLRFVGRSGRGKSFTLTITVFTGPPQVA

T.r.-Runx1 TLVTVMAGNDENYSAELRNATAAIKNQVARFNDLRFVGRSGRGKSFTLTITVFTSPPQVA

T.r.-Runx2 TVVTVMAGNDENYSAELRNASGVMKNQVARFNDLRFVGRSGRGKSFTLTITVFTNPPQVA

T.r.-Runx3 TLVTVMAGNDENYSAELRNASAVMKNQVARFNDLRFVGRSGRGKSFTLTITVFTGPPQVA

T.r.-FrRunt VVVTVMAGNEENSSAELRNATATMKQGFAHFNDLRFIGRSGRGKSFTVSINVLMSPPQIA

G.a.-FrRunt VVVTVMAGNDDNNSAELRNATATMKQGYAHFNDLRFIGRSGRGKSFTLSINVLTSPPQIA

G.a.-Runx1 TLVTVMAGNDENYSAELRNATAAIKNQVARFNDLRFVGRSGRGKSFTLTITVFTNPPQVA

G.a.-Runx2 TVVTVMAGNDENYSAELRNASGVMKNQVARFNDLRFVGRSGRGKSFTLTITVFTNPPQVA

G.a.-Runx3 TLVTVMAGNDENYSAELRNASAVMKNQVARFNDLRFVGRSGRGKSFTLTITVFTGPPQVA

M.m.-Runx2 TVVTVMAGNDENYSAELRNASAVMKNQVARFNDLRFVGRSGRGKSFTLTITVFTNPPQVA

M.m.-Runx3 TVVTVMAGNDENYSAELRNASAVMKNQVARFNDLRFVGRSGRGKSFTLTITVFTNPTQVA

M.m.-Runx1 TLVTVMAGNDENYSAELRNATAAMKNQVARFNDLRFVGRSGRGKSFTLTITVFTNPPQVA

H.s.-Runx1 TLVTVMAGNDENYSAELRNATAAMKNQVARFNDLRFVGRSGRGKSFTLTITVFTNPPQVA

H.S.-Runx2 TVVTVMAGNDENYSAELRNASAVMKNQVARFNDLRFVGRSGRGKSFTLTITVFTNPPQVA

H.S.-Runx3 TVVTVMAGNDENYSAELRNASAVMKNQVARFNDLRFVGRSGRGKSFTLTITVFTNPTQVA

############################################################

250 260 270 280 290 300

=========+=========+=========+=========+=========+=========+

S.p.1 TYNRAIKVTVDGPREPR----------RPKPKDQESRLMPPPIINTGHPHPFGEINPHHP

S.p.2 TCNRAIKVTVDGPRPRRELYL------RPKSK-HDDPLCFSSIIDTGHAHSYGLVISHHD

O.d. VYHRAIKITVDGPREPRS----------EYRTQNFKSCNDYLEHRQKQLEEQGRGISFGA

C.i. TYQRAIKITVDGPREPRR----------HRQKQLEERAKGXLFPEGFHLESIRRNPTYQG

B.l. TYHRAIKVTVDGPREPRR----------HRQKLEE---QKHALSFSERLSELGLERLRHS

M.g.-RunxA TYHRAIKVTVDGPREPRR----------HRQRMEEPGIKAGGSLFPDRLCDL--DQLRRP

M.g.-RunxB TYQRAIKVTVDGPREPRR----------HRQRPEE---------HPLPFTDL--EHYCRS

S.c.-Runx1 TYHRAIKITVDGPREPRIRPNVFLCPAGHRQKLEE-QSKNASLAFSERLSEL--EQLRRS

S.c.-Runx2 TYHRAIKVTVDGPREPRR----------HRQKPED-QPKVG--LFSERLSEL--ERLRQT

S.c.-Runx3 TYHRAIKVTVDGPREPRR----------HRQKLED-QSKPGT-LFSDRLSEL--ERYRQT

D.r.-Runx1 TYQRAIKITVDGPREPRR----------HRQKPDE-AVKPGALAFSE--------QLRRS

D.r.-Runx2b TYHRAIKVTVDGPREPRR----------HRPKLDD-SPKAG--LFSDRLSEL--ERIRQT

D.r.-Runx2a TYHRAIKVTVDGPREPRR----------HRQKLED-PPKPP--LFSERLSEL--ERLRQT

D.r.-Runx3 TYHRAIKVTVDGPREPRR----------HRVKPDD-PHK----QFSDRLSDI--ERFQRA

T.r.-Runx1 TYQRAIKITVDGPREPRR----------HRQKMD--EVKPGALAFSERLSEL--EHLRRS

T.r.-Runx2 TYHRAIKVTVDGPREPRR----------HRQKLED-PPKAG--LFSDRLSEL--ERMR--

T.r.-Runx3 TYHRAIKVTVDGPREPRR----------HRVKVED-SQKM---QFSDRLSEI--ERYQR-

T.r.-FrRunt TLQKAIKVTVDGQRQPRR-----------QRQKE----------------------VKSG

G.a.-FrRunt TVHRAIKVTVDGQRLPRR-----------TESWE----------------------FSG-

G.a.-Runx1 TYQRAIKITVDGPREPRR----------HRQKMD--EVKPGTLAFSERLTEL--EQLRRS

G.a.-Runx2 TYHRAIKVTVDGPREPRR----------HRQKLED-PPKSG--LFSDRLSEL--ERMR--

G.a.-Runx3 TYHRAIKVTVDGPREPRR----------HRVKIED-PQKM---QFSDRLSEI--ERYQR-

M.m.-Runx2 TYHRAIKVTVDGPREPRR----------HRQKLDD-S-KPS--LFSDRLSDL--GRIPHP

M.m.-Runx3 TYHRAIKVTVDGPREPRR----------HRQKIED-QTKA----FPDRFGDL--R-----

M.m.-Runx1 TYHRAIKITVDGPREPRR----------HRQKLDD-QTKPGSLSFSERLSEL--EQLRRT

H.s.-Runx1 TYHRAIKITVDGPREPRR----------HRQKLDD-QTKPGSLSFSERLSEL--EQLRRT

H.S.-Runx2 TYHRAIKVTVDGPREPRR----------HRQKLDD-S-KPS--LFSDRLSDL--GRIPHP

H.S.-Runx3 TYHRAIKVTVDGPREPRR----------HRQKLED-QTKP----FPDRFGDL--ERL---

################## ################################

310 320 330 340 350 360

=========+=========+=========+=========+=========+=========+

S.p.1 NHHIGRQ-----------QSYQNQGRMPRSYPLSPTSGSYDNIQHQGQASKPWSYYNPYQ

S.p.2 N-QIGRQ-----------QPFVNQGWMPGSYPLTSTSCD----------NQPEFHTNHYT

O.d. TLGEMER-----------LRTAVAG-GYADSMFSSFGTSDFRSAAAAGWPYSTQS-----

C.i. QVDPSRM-----------VASGTGGWAYPQPQFPYLTPSQHAAAAARAQSVARAGNVAGL

B.l. HIAQPLP-------PAIRTPLIEAPPGHHPPPSAYTPGNPQIQATQDGASRSPPSSWPYQ

M.g.-RunxA TVRLSPHLG---------GGGSR-------------------------------------

M.g.-RunxB SIRHGTP-----------TSGHQTPLNPSSHYTIPTGTEPGTCGE--------------S

S.c.-Runx1 AMRLSP----HHPTT---APNPR--PSLNPTTAFNPQAQSQIQDTRHIQPSPPWTYDQSY

S.c.-Runx2 AMRVGAS-----------TQSPRPSLNPAPAPFSPQATSQIAG-----------------

S.c.-Runx3 TMRVGPS-----------TPSPR-----PLGVPT--------------------------

D.r.-Runx1 AMRCSP----HHGP----APNTR--PTLNTPPFGSPAHSQIP-DSRQMQTSPSWSYEQSY

D.r.-Runx2b TMRVTVP-----------TQTPRPSLSS-PNSYTPQGQTQITDPRQAQSSPPWSYDQTYP

D.r.-Runx2a TMRVAVQ-----------TQSPRPSLNATPNSFNPQGQTQISDPRQAQSSPPWSYEQPYP

D.r.-Runx3 SLRMNPG-----------NGTTRP-HQTHYSPSAPTQIP--------------------G

T.r.-Runx1 SMRVTPPHHHHHHHHQAASANSRQSAVLNAATFSSPPHTQLTADSRQMQSSPSWSYDQSY

T.r.-Runx2 -VRVAVP-----------TQGPRPTLNTVANSFNPQGQTQITDPRQSQSSPPWSYDQTYQ

T.r.-Runx3 SMRIGTA-----------NNNPRPIHQAHLSTTQ-------------------------A

T.r.-FrRunt AFRPGTC-----------------------------------------------------

G.a.-FrRunt -YLATAL-----------------------------------------------------

G.a.-Runx1 SMRGTPPHHHHHQH----SANTRQAAALNSATFSSPTHAQITADSRQMQPSPSWSYDQSY

G.a.-Runx2 -VRVAIP-----------TQGPRPTLNTGAASFNPQGQTQITDPRQSQSSPPWSYEQTYP

G.a.-Runx3 SMRIGPV-----------NNNPRPIHQTHLSTTQ-------------------------A

M.m.-Runx2 SMRVGVP-----------PQNPRPSLNSAPSPFNPQGQSQITDPRQAQSSPPWSYDQSYP

M.m.-Runx3 -MRVTPS-----------TPSPRG----SLSTTS--------------------------

M.m.-Runx1 AMRVSP----HHPAP---TPNPR--ASLNHSTAFNPQPQSQMQDARQIQPSPPWSYDQSY

H.s.-Runx1 AMRVSP----HHPAP---TPNPR--ASLNHSTAFNPQPQSQMQDTRQIQPSPPWSYDQSY

H.S.-Runx2 SMRVGVP-----------PQNPRPSLNSAPSPFNPQGQSQITDPRQAQSSPPWSYDQSYP

H.S.-Runx3 RMRVTPS-----------TPSPRG----SLSTTS--------------------------

###

370 380 390 400 410 420

=========+=========+=========+=========+=========+=========+

S.p.1 SSVAQLSDTSILSAQIKTEPTELALLGQQNSTLQQYPKPDSLYPTSITRS----SEVQDP

S.p.2 PSVQQSTGNPILPPQIKTEPPELDLVAVQQ---------------------------NQP

O.d. -PLRTFSQHYGSTISPTAGNQPS-------------------------------------

C.i. GPAGSITPPGGLNMIKREESHGSGQ-----------------------------------

B.l. PYQPFMGAPMPPPAMPPQTTAAATIERISPEMAGRLPTTMP-------------------

M.g.-RunxA ---APLTGPGAFNAQPQIQLQGPG------------------------------------

M.g.-RunxB RHLSAIPSLPDPSHYGDSRLHCPG------------------------------------

S.c.-Runx1 PYLGQIPAPSVHPATPISPGRTGGMQAITAELSSRLSGAS--------------------

S.c.-Runx2 -------ATDFHFTD----SRQFG------------------------------------

S.c.-Runx3 HFSPQAQTQMQGTS----DISPFA------------------------------------

D.r.-Runx1 PYLGPISTPAVHPTTPISPNRT-----ALH--CPELTA----------------------

D.r.-Runx2b SYLSPMASPSVHSTTPLSSTGATGQPGHGSALHQRCA-----------------QTSSGS

D.r.-Runx2a PYLSQMTSPSIHSTTPLSSTRATGLPTISDVPRRLS----------------------GT

D.r.-Runx3 LWPDQIDPPTLKTCKVEDDLR-WT-----------------------------------S

T.r.-Runx1 PYLGQIATPTMHTANPLSPSRS-----SLGDLSSRLTGP---------------------

T.r.-Runx2 SYLSPMASPSVHSTTPLSSSRGTGLPAISDVPRR----------------------LPGS

T.r.-Runx3 LWQEQMDTPSLKTCKVEEDLRPWPG----------------------------------T

T.r.-FrRunt -STASADCRSFSSSLWTSEPSFLG------------------------------------

G.a.-FrRunt -HHRVCRVESFSS-RWTNEPSLL-------------------------------------

G.a.-Runx1 PYLGQITTPSVHTSNPLSPGRS-----SLSDLSSRLAG----------------------

G.a.-Runx2 SYLSPMASPSVHSTTPLSSSRGTGLPAISDVPRR----------------------LPGS

G.a.-Runx3 LWQEQMDTPALKTCKVEEDLRPWPG----------------------------------T

M.m.-Runx2 SYLSQMTSPSIHSTTPLSSTRGTGLPAITDVPRRISDDDTATSDFCLWPSSLSKKSQAGA

M.m.-Runx3 HFSSQAQTPIQGSS----DLNPFS------------------------------------

M.m.-Runx1 QYLGSITSSSVHPATPISPGRASGMTSLSAELSSRLSTAP--------------------

H.s.-Runx1 QYLGSIASPSVHPATPISPGRASGMTTLSAELSSRLSTAP--------------------

H.S.-Runx2 SYLSQMTSPSIHSTTPLSSTRGTGLPAITDVPRRIS----------------------GA

H.S.-Runx3 HFSSQPQTPIQGTS----ELNPFS------------------------------------

#################

430 440 450 460 470 480

=========+=========+=========+=========+=========+=========+

S.p.1 RFVYPSTPAAVSSVSFTPSSMSVLSSGVESPRTILPMTPNPFPLSS---QDIFSSSSTAT

S.p.2 QFIYPTQPGDTPFYPSQSSSMSGVSPGEDPQRTLLPMTPNQLPLPSQPAQDVFSSST-AI

O.d. ----------TTPKSDKASSHDNTSPFPRVPDMSYP------------------YSTAMN

C.i. ----LSGGNGISPPCTTGNGTGNESLTIRFSEVHLEGRFGNRSFVYPSGAGFPGYDSSML

B.l. EVISQRFPGDIPLRFPADSQFSYPDLRFSDPRLSDPRLLYPAAGA--------TGFTAYS

M.g.-RunxA -ELGALA----------AGLSSLSDSRFSDPRMHYPGAVP---------------AFHYP

M.g.-RunxB PTLPLYP------APGPPGAGSLDIPRYTYLPPPYPTNAQPQN----------HQGSPYQ

S.c.-Runx1 -DLTAFVDPRMSIDRQFSALPSLSDGRFTDPRMHYPGP------------------FPYT

S.c.-Runx2 -------------------LSSLTESRFPNPRMHYP------------------AAFTYT

S.c.-Runx3 -DHRQFE-------RQFPGLSSLTESRFSDPRMNYPGAMSA-------------AAFTYA

D.r.-Runx1 -----FTDPRVGLERSFPSLPSLPDGRFSDPRVP-YPTG----------------AFTYT

D.r.-Runx2b TDLSPFP---GQFERQFPAFSSLTESRFSSPRMHYP------------------ATFTYT

D.r.-Runx2a SELSPFSADPRQFERQFPSLSSLTDSRFPSPRMHYP------------------ATFTYT

D.r.-Runx3 TDLFQQR-------TSFPSLSPLTAPRFSDSHMHYP------------------SHFTYS

T.r.-Runx1 -DLAAFSDPRMSLDRSFSSLSALPDTRFSDPRVHYTPTGA---------------AFSYT

T.r.-Runx2 SDLSPFP---GQFDRQFPGLPSITESRFSSPRMHYP------------------ATFTYT

T.r.-Runx3 TDLFQQR-------TPFPSLSPLTDPRFSDPRMHYP------------------GAFPYS

T.r.-FrRunt ------------------QVTSLSSPFTPSPRMHHLP------------------TFSYA

G.a.-FrRunt -----------------------ASSFTPSPRMHHLP------------------ALAYS

G.a.-Runx1 -----NAHKHVNKEADYGKRVAL--ELLSE-----RPT-------------------QYR

G.a.-Runx2 SDLSPFP---AQFERQFPGLPPLAETRFSSPRMHYP------------------ATFTYT

G.a.-Runx3 TDLFNQR-------TSFPSLSPLTDPRFSDPRMHYP------------------GAFPYS

M.m.-Runx2 SELGPFS-DPR----QFPSISSLTESRFSNPRMHYP------------------ATFTYT

M.m.-Runx3 -DPRQFD-------RSFPTLQSLTESRFPDPRMHYPGAMS--------------AAFPYS

M.m.-Runx1 -DLTAFGDP-----RQFPTLPSISD-----PRMHYPGA------------------FTYS

H.s.-Runx1 -DLTAFSDP-----RQFPALPSISD-----PRMHYPGA------------------FTYS

H.S.-Runx2 SELGPFS-DPR----QFPSISSLTESRFSNPRMHYP------------------ATFTYT

H.S.-Runx3 -DPRQFD-------RSFPTLPTLTESRFPDPRMHYPGAMS--------------AAFPYS

#################### ####

490 500 510 520 530 540

=========+=========+=========+=========+=========+=========+

S.p.1 PVTLTSPPYLPNSPPYPLYPHLYMSSPSSQTYYDSSHLPMLPSSTRPEDKQEIK-HDNRP

S.p.2 PMTPSAN---------PIYPHLYMSTPSSQTHYGSG--SMLPSR-RPEEKRELEDKEDTS

O.d. PAASATSSAG--YG-------AFDSSQLMSNYLASTQLAAGG----LGAVNYLGSAYS--

C.i. PAGGHNQSSNNPYGTPYLYPAALYPGNTLSSYNSVGQNYDGGSS--VGSEGDISSTMSPG

B.l. SGPTTTMSMLPTSLTSPRYLPMSPPGFPSLTSAPGGFVTSPNSP--PRQLGNYSPGSPPY

M.g.-RunxA TAAPTSAAPL---ALSMPAVATAAAARYHTYHLPPPCPGAT-----QNQVPPFQSTSTPY

M.g.-RunxB AASASFSSTS---TPFQASPAHFSASPTPFINHGPFFQCSAP-F--GSGSSSFQAGPAPY

S.c.-Runx1 PTPVTTG------IGIGMS--AMTTAARYHTYLPPPYPGS---A--QGQTGPFQTSSSPY

S.c.-Runx2 PTPVTTG------MS----LGMSAATHYH-TYLPPPYPGSS-----PNQSGPFQTSSAP-

S.c.-Runx3 TTPSTGG------IGGISMTGMAAAARYH-TYLPPPYPGSA-----QNQSGPFQTSVSPY

D.r.-Runx1 PTPVTS---A---IGIGMS-AMSSPAGRYHTYLPPAYPAG-S-S--QAQAGAFQASSSPY

D.r.-Runx2b PTPVTTG------MS----LG---SAHYH-TYLPPPYPGST-----QSQSGPFQSSSTP-

D.r.-Runx2a PTPVTSG------MS----LGMSTTTHYH-TYLPPPYPGST-----QNQSGPFQTSSTP-

D.r.-Runx3 ANPSSTG------IGGLSVAGMPTSSRYH-TYLPPPYPGN------QNQNSHFQTNSSPY

T.r.-Runx1 PSHNPVSNGT---LGITMATAMATTPTGRYTYLPPPYPANTP-H--QAQNGPFQSPSSAY

T.r.-Runx2 P-PVTTG------MS----LG---SAHYH-TYLPPPYPGST-----QSQSTPFQTSSTP-

T.r.-Runx3 TNTSSTG------ISGLSMS---ASSRYH-TYLPPPYTNN------QTQN--FQSNS---

T.r.-FrRunt TQPTTYT-----------------------SYLSSPPPPP------LNHSSSFQPGS---

G.a.-FrRunt TQPTGYT-----------------------SFLSAPPPPP------LSHSGPFQPSS---

G.a.-Runx1 AQQN---------IRLGVNSGISSVACWVCVYVKLNHKGPKL-K--TGMNSKIPD-SRLS

G.a.-Runx2 P-PVTSA------MS----LG---SAHYH-TYLPPPYPGST-----QTQSGPFQTSSTP-

G.a.-Runx3 TNTSSTG------ISGLSMS---ASSRYH-TYLPPPYSNN------QSQN--FQSNP---

M.m.-Runx2 P-PVTSG------MS----LGMSATTHYH-TYLPPPYPGSS-----QSQSGPFQTSSTP-

M.m.-Runx3 ATPSGTS------LGSLSVAGMPASSRFHHTYLPPPYPGAP-----QSQSGPFQANPAPY

M.m.-Runx1 P-PVTSG------IGIGMS--AMSSASRYHTYLPPPYPGS---S--QAQAGPFQTGSPSY

H.s.-Runx1 PTPVTSG------IGIGMS--AMGSATRYHTYLPPPYPGS---S--QAQGGPFQASSPSY

H.S.-Runx2 P-PVTSG------MS----LGMSATTHYH-TYLPPPYPGSS-----QSQSGPFQTSSTP-

H.S.-Runx3 ATPSGTS------ISSLSVAGMPATSRFHHTYLPPPYPGAP-----QNQSGPFQANPSPY

##### ##############################

550 560 570 580 590 600

=========+=========+=========+=========+=========+=========+

S.p.1 ----GEGIPHIPHP--EMSLTVALNYGSHPQSQIELNTARTMGQSMGG-MHNSGVALMQQ

S.p.2 HGISVEIITNLPHP----YPDMPLSYGGHPQNQIELDRERTTGIQSGPGRMTKAIVIVNC

O.d. NSMGSSLP---SVPAVTSS----------PATTLPSLPTVTSPHSITP----GSTIAAAA

C.i. NSSGGARDGHDGSQNVDNG----------AANLINSAVGLHGQRWDNS----NGVVAAAL

B.l. GIYHHLYGGSYQYPILPGGSG-TPQGPREPSILAATSN---SQTQKP--------QLLPA

M.g.-RunxA HLYYGASSGSYQFPMVAGS------GERSP--------------GRG--------VLLSG

M.g.-RunxB PLYY-SHSASYQLSIIEGEDRPPVHHHQTFPTMGAPRLVVPGVAGSS------SVLLTPG

S.c.-Runx1 HLYYGTSAGSYQFSMMTGGD-------RSPPRILPPCTN--ASTGSS--------LLNPS

S.c.-Runx2 YLYYGTSSGSYQFSMVA-------GGERSPSRMLPPCT--SASTGST--------LLNPN

S.c.-Runx3 HLYYGASAGSYQFSMVAGG-------ERSPTRMLPSCT---SASSGS-------TLMNPN

D.r.-Runx1 HLYYSSAAGSYQFSMMPSGGA--AAGERSPPRILP-CTN--ASTGSA--------LLHPS

D.r.-Runx2b YLYYGASSGSYQFSMVPGSM--VPGGERSPTRMMPPCT--SASTGTS--------LVNPN

D.r.-Runx2a YLYYGASSGSYQFPMVP-------GGDRSPSRMLPPCT--SASTGST--------LINPN

D.r.-Runx3 HLYYGTGSGSYQFSMIPTGGS-GSGGDRSPTRMLTSCTAAGGAAGNTG----NNNLINAN

T.r.-Runx1 HLYYSTATGSYQFPMMAGGGG--RGDSRSPPRILPPCTN--ASTGSP--------LLHPS

T.r.-Runx2 YLYYGASSGSYQFSMVP-------GGDRSPSRMIPPCT--SASTGTT--------LVNPN

T.r.-Runx3 YLYYGTGSGSYQFSMVAPG---NTGGERSPTRLLS----CSGATGTGG----ANSLMNPG

T.r.-FrRunt -FYYGQNQQFYSMAEDRN--------------------------------------VVTA

G.a.-FrRunt -FYYGPSQTYQTAGEERN--------------------------------------VVTT

G.a.-Runx1 CRKYAVPSGTFSISRSS--------DSRSPPRILPPCTN--ASTGSS--------LLHPS

G.a.-Runx2 YLYYGASSGSYQFSMVP-------GGDRSPSRMIPPCT--SASTGTS--------LVNPN

G.a.-Runx3 YLYYGTGSGSYQFSMVAQG---NTGGERSPTRLLS----CSGATGPGG----TNGLMNQG

M.m.-Runx2 YLYYGTSSASYQFPMVP-------GGDRSPSRMVPPCT--TTSNGST--------LLNPN

M.m.-Runx3 HLFYGASSGSYQFSMAAAG-----GGERSPTRMLTSCP-SGASVSAG-------NLMNPS

M.m.-Runx1 HLYYGASAGSYQFS-MVGGE-------RSPPRILPPCTN--ASTGAA--------LLNPS

H.s.-Runx1 HLYYGASAGSYQFS-MVGGE-------RSPPRILPPCTN--ASTGSA--------LLNPS

H.S.-Runx2 YLYYGTSSGSYQFPMVP-------GGDRSPSRMLPPCT--TTSNGST--------LLNPN

H.S.-Runx3 HLYYGTSSGSYQFSMVAGSS---SGGDRSPTRMLASCTSSAASVAAG-------NLMNPS

############### #################### #####

610 620 630 640

=========+=========+=========+=========+=====

S.p.1 HRNHSPVTNISPMQQSMGQMNINTTVGHLPTVDDSRKEDVWRPY-

S.p.2 HALKVSVGLEYSKEKGDGAHNV--------CVDESNNQGSYYL--

O.d. A------------MPHHPIHTPQLPPVASSAAHAAVSALSWATDN

C.i. EXVEAASRGQRDNMDHHSHHHHQVARXAGVNTSGILHEDVWRPYW

B.l. QEKEASS---QPGNGNHGNRQQDEQMDTQPGGSPEKRETVWRPY-

M.g.-RunxA LPGTGET----DAGTGHRSSP----------PGSRLDETVWRPY-

M.g.-RunxB LSSSDGDGG-VEGDGSNENSPSSD--LVEPHQARLDHETVWRPY-

S.c.-Runx1 LPNQSDVV---DADGSHSNSPTN------MSTTGRLEEAVWRPY-

S.c.-Runx2 LPNQNDG---VDADGSHSNSPTV------LTSTGRIDESVWRPY-

S.c.-Runx3 LANQNDV---VDADGSQSNSPTA------MSTTGRMDESVWRPY-

D.r.-Runx1 LPNQSEGV--VEAEGSHSSSPTS------MS-----VEAVWRPY-

D.r.-Runx2b LPVQADGSGGVEGDGSHSNSPTL------LNPAGRMDEGVWRPY-

D.r.-Runx2a LPNQTDG---GEADGSHSSSPTV------LNSSGRMDESVWRPY-

D.r.-Runx3 LGNQSDG---VDADGSHSNSPTA------MSASTRIDESVWRPY-

T.r.-Runx1 LPNQNDGVG-VEVESSHSSSPTN------MAA----AEAVWRPY-

T.r.-Runx2 LPSQTEG--AVDGDGSHSNSPTI------LNPGGRMDEAVWRPY-

T.r.-Runx3 LNAQSEG---VEADGSHSNSPTA------MNASGRLDESVWRPY-

T.r.-FrRunt LTNYIEG-------ACLSMR---------------GEEPVWRPY-

G.a.-FrRunt LANYIEG-------ACLPMR---------------GEEPVWRPY-

G.a.-Runx1 LPNQNGGVG-VEVESSHSSSPTN------MAA----AEAVWRPY-

G.a.-Runx2 LPSQSEG--GVDADGSHSNSPTV------LNPGGRMDEAVWRPY-

G.a.-Runx3 LNNQSEG---VEADGSHSNSPTA------MSASGRLDESVWRPY-

M.m.-Runx2 LPNQNDG---VDADGSHSSSPTV------LNSSGRMDESVWRPY-

M.m.-Runx3 LG-QADG---VEADGSHSNSPTA------LSTPGRMDEAVWRPY-

M.m.-Runx1 LPSQSDVV---ETEGSHSNSPTN------MPP-ARLEEAVWRPY-

H.s.-Runx1 LPNQSDVV---EAEGSHSNSPTN------MAPSARLEEAVWRPY-

H.S.-Runx2 LPNQNDG---VDADGSHSSSPTV------LNSSGRMDESVWRPY-

H.S.-Runx3 LGGQSDG---VEADGSHSNSPTA------LSTPGRMDEAVWRPY-

###################### ##########

**Fig. S1:** Alignment (ClustalW, BioEdit: http://www.mbio.ncsu.edu/BioEdit/bioedit.html) of newly detected *Runt* genes in hagfish (MgRunxA and B, DQ990008, DQ990009) and dogfish (*ScRunx1-3*, DQ990010, DQ990012, DQ990014) with other deuterostome *Runt* genes. The conserved sequence blocks used for the phylogenetic analysis are underlined with #. Parameters used with Gblocks 0.91b were: Minimum number of sequences for a conserved / flanking position: 15/15; Maximum number of contiguous nonconserved positions: 8; minimum length of a block: 5; allowed gap positions: all. 338 (52%) of the original 645 alignment positions were used in the phylogenetic analysis.Abbreviations: B.l.: *Branchiostoma lanceolatum*; C.i.: *Ciona intestinalis*; D.r.: *Danio rerio*; G.a.: *Gasterosteus aculeatus*; H.s.: *Homo sapiens*; M.m.: *Mus musculus*; M.g.: *Myxine glutinosa*; O.d.: *Oikopleura dioica*; S.p.: *Strongylocentrotus purpuratus*; S.c.: *Scyliorhinus canicula*; T.r.: *Takifugu rubripes*.
