# Supplementary material for: Evolution of a Core Gene Network for Skeletogenesis in Chordates
Source: PLoS Genet. 2008 Mar 21;4(3):e1000025. doi: 10.1371/journal.pgen.1000025 (PMC2265531; doi:10.1371/journal.pgen.1000025)
Supplement: Figure S2 — Synteny Analysis. A search for cross-species conserved gene orders was performed as previously described [1]. We compared a larger contig of the FrRunt locus (Ensemble: Scaffold 39) than previously analyzed (Ensemble: Scaffold 835[2]) to the zebrafish genome and detected a synteny region between the 3′ genomic region of the FrRunt gene and chromosome 1 of zebrafish comprising Fstl1 and Gja5 (A). Furthermore we detected in the stickleback (G. aculeatus) genome a FrRunt orthologous gene with a genomic environment almost identical to the FrRunt gene locus (B). The gene orthologous to Clic 5 located 3′ of Runx2a in the zebrafish genome was found by Blast searches on group 1 in the stickleback genome. Together these results suggest that a translocation between a region of the 3′ end of the FrRunt locus and chromosome 1 had occurred in the common stem species of pufferfish and stickleback. (0.05 MB DOC) [file pgen.1000025.s002.doc]

(A)

Fugu (FUGU4) - *Danio rerio* (__ZFISH6.45)

**SINFRUG00000162875 (FrRunt)**

SINFRUG00000141701

SINFRUG00000141696

Fugu -

(scaffold 39)

*D. rerio* - (17)

SINFRUG00000141707 (Fstl1)

SINFRUG00000129429 (Gja5)

*D. rerio* - (1)

ENSDARG00000015559 (Fstl1)

ENSDARG00000015076

ENSDARG00000036688 (Gja5)

SINFRUG00000161421

SINFRUG00000161417

SINFRUG00000141690

SINFRUG00000148702

ENSDARG00000032589

ENSDARG00000024936

ENSDARG00000025241

ENSDARG00000060310

*D. rerio* - (12)

ENSDARG00000040261 (Runx2a)

ENSDARG00000002776 (Supt3h)

ENSDARG00000043797 (Cdc5l)

B)

Fugu - *Gasterosteus aculeatus* (BROADS1.45)

**SINFRUG00000162875 (FrRunt)**

SINFRUG00000141701

SINFRUG00000141696

Fugu -

(scaffold 39)

SINFRUG00000141707

SINFRUG00000129429

SINFRUG00000161421

SINFRUG00000161417

SINFRUG00000141690

SINFRUG00000148702

ENSGACG00000011699

ENSGACG00000011701

ENSGACG00000011721

ENSGACG00000011726

ENSGACG00000011733

ENSGACG00000011818 -

ENSGACG00000011822

ENSGACG00000011832

ENSGACG00000011835

ENSGACG00000011843

*G. aculeatus*

(groupVI)

**Fig. S2:** A search for cross-species conserved gene orders was performed as previously described [1]. We compared a larger contig of the *FrRunt* locus (Ensemble: Scaffold 39) than previously analyzed (Ensemble: Scaffold 835[2]) to the zebrafish genome and detected a synteny region between the 3´ genomic region of the *FrRunt* gene and chromosome 1 of zebrafish comprising *Fstl1* and *Gja5* (A)*.* Furthermore we detected in the stickleback (*G. aculeatus*) genome a *FrRunt* orthologous gene with a genomic environment almost identical to the *FrRunt* gene locus (B). The gene orthologous to *Clic 5* located 3´ of *Runx2a* in the zebrafish genome was found by Blast searches on group 1 in the stickleback genome. Together these results suggest that a translocation between a region of the 3´end of the *FrRunt* locus and chromosome 1 had occurred in the common stem species of pufferfish and stickleback.

Reference List

1. Rödelsperger C., Dieterich C. (2007) Two Graph-based Approaches for Finding Cross-species Conserved Gene Orders. Proceedings of the German Conference on Bioinformatics 115: 163-173.

2. Glusman G, Kaur A, Hood L, Rowen L (2004) An enigmatic fourth runt domain gene in the fugu genome: ancestral gene loss versus accelerated evolution. BMC Evol Biol 4: 43.
