# Supplementary material for: Evolution of a Core Gene Network for Skeletogenesis in Chordates
Source: PLoS Genet. 2008 Mar 21;4(3):e1000025. doi: 10.1371/journal.pgen.1000025 (PMC2265531; doi:10.1371/journal.pgen.1000025)
Supplement: Table S1 — Dogfish Primers. Primers employed to amplify and analyze the expression of Runt genes in dogfish. PA: Primary amplification, RA: Reamplification. (0.08 MB DOC) [file pgen.1000025.s003.doc]

Table S1: Primers employed to amplify and analyze the expression of *Runt* genes in dogfish. PA: Primary amplification, RA: Reamplification.

| ***S. canicula* (*ScRunx1-3*)** | | |
| --- | --- | --- |
| ***Gene name*** | **Primer name** | **Primer sequence** |
| Amplification of conserved *ScRunt1-3* fragments | Sc1-up- | accatggcwtcsaacagcata |
|  | Sc2-up- | accatggcwtcsaacagcatatt |
|  | Sc3-up | cac tgg mgv tgc aay aar ac |
|  | Sc4-up | GTC ACM GTG GAY GGR CC |
|  | Sc5-up | GGC MGA GCN AAR AGY TTC AC |
|  | Sc6-up | TTC AAY GAC CTG AGN TTY GT |
|  | Sc7-up | gcc atc ast gac gtk ccy ag |
|  | Sc8-up | ack gag agc cgy ttc tcc a |
|  | \| Sc9-up \| \| --- \| | cca cct tya cct aca ccc crc c |
|  | Sc1-low | GGT CCR TCC CAN GTG AY |
|  | Sc2-low | CAG CGT GAA RCT YTT NCC TC |
|  | Sc3-low | gcg ccr tag tag tga tat gg |
|  | Sc4-low | TCA GTA CGG CCK CCA GAC |
|  | Sc5-low | tca ata agg ccg cca gac dga ytc |
| *ScRunx1*-RACE | 3’-RACE-PA | GGACCTACGACCAGTCGTACCCCTATCTTG |
|  | 3’-RACE-RA | GCGAACAGGTGGGATGCAGGCAATCACAG |
|  | 5’-RACE-PA | CTGTGATTGCCTGCATCCCACCTGTTCGC |
|  | 5’-RACE-RA | CAAGATAGGGGTACGACTGGTCGTAGGTCC |
| *ScRunx2*-RACE | 3’-RACE-PA | tgccgatcacccggcagagttggtccggac |
|  | 3’-RACE-RA | caacacagagtcctcgtccatctctcaat |
|  | 5’-RACE-PA | ctcatcattgccggccatgacagtgaccaca |
|  | 5’-RACE-RA | ggctcctggtgagggatcatcccgctcac |
| *ScRunx3-*RACE | 3’-RACE-PA | CTCAAACACAGATGCAGGGCACCTCGGAC |
|  | 3’-RACE-RA | GTCTGCTGCTGCCTTCACCTACGCCACCA |
|  | 5’-RACE-PA | TGGTGGCGTAGGTGAAGGCAGCAGCAGAC |
|  | 5’-RACE-RA | GTCCGAGGTGCCCTGCATCTGTGTTTGAG |
| qRT-PCR | up-18S-Taq | ACCACATCCAAGGAAGGCAG |
|  | low-18S-Taq | CCGAGTCGGGAGTGGGTAAT |
|  | up-*Runx1*-Taq | CGTACCCCTATCTTGGCCAGAT |
|  | low-*Runx1*-Taq | TGGAAAGTTCTGCTGTGATTGC |
|  | up-*Runx2*-Taq | CCTCACAGAAAGCCGATTTCCT |
|  | low-*Runx2*-Taq | GCTGCAGACATACCCAATGACA |
|  | up-*Runx3*-Taq | TCGGCAATTTGAGCGTCAGTT |
|  | low-*Runx2*-Taq | GAAGGCAGCAGCAGACATAGCA |
| *ScRunx1* ISH probe | up | aagttggaagagcagagtaaaaat |
|  | low | TCATAGTCAATATGGCCGCCA |
| *ScRunx2* ISH probe | up | aagcctgaagaccaacccaaagtc |
|  | low | TCAGTATGGTCTCCAGACAGACTC |
| *ScRunx3* ISH probe | up | aagcttgaagaccaatcaaaacc |
|  | low | CTAATAAGGCCGCCAGACTGA |
