# Supplementary material for: Evolution of a Core Gene Network for Skeletogenesis in Chordates
Source: PLoS Genet. 2008 Mar 21;4(3):e1000025. doi: 10.1371/journal.pgen.1000025 (PMC2265531; doi:10.1371/journal.pgen.1000025)
Supplement: Table S2 — Hagfish Primers. Primers employed to detect Runt genes and analyze Runt gene expression in hagfish. PA: Primary amplification, RA: Reamplification. (0.07 MB DOC) [file pgen.1000025.s004.doc]

Table S2: Primers employed to detect *Runt* genes and analyze *Runt* gene expression in hagfish. PA: Primary amplification, RA: Reamplification.

| ***Gene name*** | **Primer name** | **Primer sequence** |
| --- | --- | --- |
| *Runt* domain of *MgRunxA* and -*B* | PA-up | CACTGGMGVTGCAACAARAC |
|  | PA-low | TCAGTACGGCCKCCAGAC |
|  | RA-up | TTCAACGACCTNCGNTTYGT |
|  | RA-low | GGNGGSAGGTANGTGTGGTAG |
| *MgRunxA*-RACE | 3´-RACE-PA | GAGCTACGGAATGCATCCGCAGTG |
|  | 3´-RACE-RA | CAAGAACCAAGTCGCAAGGTTCAAC |
|  | 5´-RACE-PA | gttgaaccttgcgacttggttcttg |
|  | 5´-RACE-RA | cactgcggatgcattccgtagctc |
| *MgRunxB*-RACE | 3´-RACE-PA | caaggtgctgtgcctcgtggacgtg |
|  | 3´-RACE-RA | gacggtaccgaggtgggcgttctggc |
| Inverse PCR to amplify  The 5´end of *MgRunxB* | PA-up | CGGACAGTTTGTTCGGCGTCAGGGTGTTC |
|  | PA-low | CGTCCGAACGGACAGTCCAAACTTCTTGTG |
|  | RA-up | TTCGAGGGTGGCGTGAACCTCCTGGTGT |
|  | RA-low | CCGTTCTACCTTCGCATTGGCGCTGC |
| qRT-PCR | 18S-up | GGCTCGAAGGCGATCAGATA |
|  | 18S-low | TCGGCATAGTTTACCGTCGAA |
|  | *RunxA*-up | CACCGTTTCAGAGCACTTCCA |
|  | *RunxA*-low | ACGAAGCCCCGTAGTAGAGATG |
|  | *RunxB*-up | CCCAACCCCCTTCATTAACC |
|  | *RunxB*-low | AAAGGCGCGGAGCATTG |
| *MgRunxA* ISH probe | up | GGATCAACTGAGGCGTCCTA |
|  | low | CTGTTTCATCCAAGCGACTG |
| *MgRunxB* ISH probe | up | caggtacgtgtggggagtct |
|  | low | gaggaccggatcagatgaaa |
