# Supplementary material for: Evolution of a Core Gene Network for Skeletogenesis in Chordates
Source: PLoS Genet. 2008 Mar 21;4(3):e1000025. doi: 10.1371/journal.pgen.1000025 (PMC2265531; doi:10.1371/journal.pgen.1000025)
Supplement: Table S3 — Amphioxus Primers. Primers employed to analyze Sox9, Hedgehog and Runt genes in lancelets. (0.08 MB DOC) [file pgen.1000025.s005.doc]

Table S3: Primers employed to analyze *Sox9*, *Hedgehog* and *Runt* genes in lancelets.

| *B. lanceolatum* (*BrlRunt, BrlSox9 & BrlHh*) | | |
| --- | --- | --- |
| ***Gene name*** | **Primer name** | **Primer sequence** |
| *Sox9* amplification | up | AGGACGTGATTCGCCTGCAGTTCC |
|  | low | TCGGCGTTGTGCAGGTGAGGATACTG |
| *Runt* ISH probe | up | ATGCTGATTCCCACCCCTT |
|  | low | ttagtacgggcgccagact |
| *Hh* ISH probe | up | cagtgggagggggtaaaact |
|  | low | agtgcaccccctcctgtaat |
| *Runt* qRT-PCR | 18S-up | CGTCCCTGCCCTTTGTACA |
|  | 18S-low | ACTGGCCTCACTAAACCATTCAA |
|  | *Runt*-up | AAGAGGGAATTCGAACGTCCTT |
|  | *Runt*-low | CAAATGGTTTTTGCACAACGAT |
| *Sox9* qRT PCR | BlSox9-up | CCACACCATGAAGGCGTTC |
|  | BlSox9-low | GTCCAAGTCGAAGCCCCAC |
| Cloning of *BlRunt* into pVAX1 (Luziferase-assays) | BlRunt-vax-up | GATAAGCTTGCCACCATGCTGATTCCCACCCCTT |
|  | BlRunt-vax-low | GATCTCGAGTTAGTACGGRCGCCAGACTG |
| Cloning of *BlRunt* into  RCAS vector (EMSA) | BlRuntRcas-up | GACGAAGACGACCATGCTGATTCCCACCCCTT |
|  | BlRuntRcas-low | GCAGAATTCTTAGTACGGRCGCCAGACTG |
| *B. floridae* (*BfRunt*, *Hh* promoter) | | |
| *Runt* ISH probe | *Runt*-Exon1-up | ctgtggtggtccatcccggcaggtgc |
|  | *Runt*-Exon1-low | tacgaaagggatccaagtcaaaggcctg |
|  | *Runt-*Exon2-up | ctgtggccgcaagccttgtaa |
|  | *Runt-*Exon2-low | cctcgtccagagcgtcccacgaag |
| *Hedgehog promoter fragments* | P1866-up | GATCTCGAGCGCATACAATAACCCGCTTT |
|  | P1708-up | GATCTCGAGCCACCAGAAGCGTTTAAGGA |
|  | P1267-up | GATCTCGAGAAGATCGTTCACGGTTTTGC |
|  | Promoter-low | GATAAGCTTAGTACCCCCGCCATAACTCT |
