# Supplementary material for: Evolution of a Core Gene Network for Skeletogenesis in Chordates
Source: PLoS Genet. 2008 Mar 21;4(3):e1000025. doi: 10.1371/journal.pgen.1000025 (PMC2265531; doi:10.1371/journal.pgen.1000025)
Supplement: Table S4 — EMSA Oligos. Oligos employed for the electrophoretic mobility shift assays. (0.07 MB DOC) [file pgen.1000025.s006.doc]

Table S4: Oligos employed for the electrophoretic mobility shift assays.

| **Name** | **Sequence** |
| --- | --- |
| R5+R6-5’ | ATGATTGTAACCGCAAAACCGCAGCAAA |
| R5+R6-3’ | AGGTTTGCTGCGGTTTTGCGGTTACAAT |
| mutR5+R6-5’ | ATGATTGTAACCGCAAAAAAGAAGCAAA |
| mutR5+R6-3’ | AGGTTTGCTTCTTTTTTGCGGTTACAAT |
| R5+mutR6-5’ | ATGATTGTAAAAGAAAAACCGCAGCAAA |
| R5+mutR6-3’ | AGGTTTGCTGCGGTTTTTCTTTTACAAT |
| mutR5+mutR6-5’ | ATGATTGTAAAAGAAAAAAAGAAGCAAA |
| mutR5+mutR6-3’ | AGGTTTGCTTCTTTTTTTCTTTTACAAT |
|  |  |
| R4-5’ | ATGGATGTTACCACAACTCCAAG |
| R4-3’ | AGGCTTGGAGTTGTGGTAACATC |
| mutR4-5’ | ATGGATGTTAAAAAAACTCCAAG |
| mutR4-3’ | AGGCTTGGAGTTTTTTTAACATC |
|  |  |
| R3-5’ | ATGGCGCACCTGCGGTCCCTCTC |
| R3-3’ | AGGGAGAGGGACCGCAGGTGCGC |
| mutR3-5’ | ATGGCGCACCTTCTTTCCCTCTC |
| mutR3-3’ | AGGGAGAGGGAAAGAAGGTGCGC |
|  |  |
| R2-5’ | ATGACCGATTTGTGGTGTGACGA |
| R2-3’ | AGGTCGTCACACCACAAATCGGT |
| mutR2-5’ | ATGACCGATTTTTTTTGTGACGA |
| mutR2-3’ | AGGTCGTCACAAAAAAAATCGGT |
|  |  |
| R1-5’ | ATGCTAGAAAACCGCAAGCCGCG |
| R1-3’ | AGGCGCGGCTTGCGGTTTTCTAG |
| mutR1-5’ | ATGCTAGAAAAAAGAAAGCCGCG |
| mutR1-3’ | AGGCGCGGCTTTCTTTTTTCTAG |
